# Chondrocyte-like cells in nucleus pulposus and articular chondrocytes have similar transcriptomic profiles and are paracrine-regulated by hedgehog from notochordal cells and subchondral bone

**DOI:** 10.3389/fcell.2023.1151947

**Published:** 2023-05-15

**Authors:** Hiroki Hagizawa, Saeko Koyamatsu, Seiji Okada, Takashi Kaito, Noriyuki Tsumaki

**Affiliations:** ^1^ Department of Tissue Biochemistry, Graduate School of Medicine and Frontier Biosciences, Osaka University, Osaka, Japan; ^2^ Department of Orthopaedic Surgery, Graduate School of Medicine, Osaka University, Osaka, Japan; ^3^ Department of Clinical Application, Center for IPS Cell Research and Application, Kyoto University, Kyoto, Japan; ^4^ Premium Research Institute for Human Metaverse Medicine (WPI-PRIMe), Osaka University, Osaka, Japan

**Keywords:** nucleus pulposus cells, chondrocytes, single-cell RNA sequencing, hedgehog, HIF-1α

## Abstract

**Objective:** The nucleus pulposus (NP) comprises notochordal NP cells (NCs) and chondrocyte-like NP cells (CLCs). Although morphological similarities between CLCs and chondrocytes have been reported, interactions between CLCs and NCs remain unclear. In this study, we aimed to clarify regulatory mechanisms of cells in the NP and chondrocytes.

**Design:** We performed single-cell RNA sequencing (scRNA-seq) analysis of the articular cartilage (AC) and NP of three-year-old cynomolgus monkeys in which NCs were present. We then performed immunohistochemical analysis of NP and distal femur. We added sonic hedgehog (SHH) to primary chondrocyte culture.

**Results:** The scRNA-seq analysis revealed that CLCs and some articular chondrocytes had similar gene expression profiles, particularly related to GLI1, the nuclear mediator of the hedgehog pathway. In the NP, cell–cell interaction analysis revealed SHH expression in NCs, resulting in hedgehog signaling to CLCs. In contrast, no hedgehog ligands were expressed by chondrocytes in AC samples. Immunohistochemical analysis of the distal end of femur indicated that SHH and Indian hedgehog (IHH) were expressed around the subchondral bone that was excluded from our scRNA-seq sample. scRNA-seq data analysis and treatment of primary chondrocytes with SHH revealed that hedgehog proteins mediated an increase in hypoxia-inducible factor 1-alpha (HIF-1α) levels.

**Conclusion:** CLCs and some articular chondrocytes have similar transcriptional profiles, regulated by paracrine hedgehog proteins secreted from NCs in the NP and from the subchondral bone in the AC to promote the HIF-1α pathway.

## 1 Introduction

Articular cartilage (AC) covers the bone ends in joints, resists loading, and reduces the friction caused by joint motion. The AC is composed of articular chondrocytes and the extracellular matrix (ECM) produced by them. The intervertebral disc (IVD) consists of nucleus pulposus (NP), annulus fibrosus, and cartilaginous endplates, which are located between the vertebral bodies and constitute the spinal column, resisting axial pressure and providing flexibility to the spinal column. The NP is composed of NP cells and the ECM produced by them.

The ECMs of the AC and NP have similar compositions: collagen fibrils that form a three-dimensional network, providing scaffolding for proteoglycan. The collagen fibrils are composed of type II, IX, and XI collagen molecules (Eyre and Muir, 1977; Roberts et al., 1991; Buckwalter and Mankin, 1998), and the proteoglycan consists of core protein aggregates onto which chains of glycosaminoglycans such as chondroitin sulfate are attached (Heinegård and Oldberg, 1989; Melrose et al., 2001). In contrast, the relative contents of collagen, proteoglycan, and water differ between the AC and NP ECMs, resulting in different tissue properties (Chen et al., 2017). NP cells are of two types: notochordal NP cells (NCs) and chondrocyte-like NP cells (CLCs). NC numbers decrease with age as they differentiate into CLCs (Choi et al., 2008; McCann et al., 2012) and become obliterated by the age of 10 years in humans.

Recently, single-cell RNA sequencing (scRNA-seq) technology has been developed to discover cell types in heterogeneous tissues and infer gene regulatory networks (Satija et al., 2015; Butler et al., 2018; Stuart et al., 2019; Hao et al., 2021). In addition, various analytical methods, such as cell–cell interaction network analysis, have been developed for downstream analysis to infer intercellular communication networks (Jin et al., 2021). Using scRNA-seq analysis, CLCs and cartilaginous endplate chondrocytes have been shown to have similar gene expression profiles in humans (Gan et al., 2021). However, precise analysis, including that of interactions between NCs and CLCs, has not been performed, probably because samples were obtained from individuals at the age of 13 years or more (Gan et al., 2021) when NCs are almost absent in the NP. We recently harvested samples from 3-year-old monkeys, performed preliminary scRNA-seq analysis, and found that chondrocytes in the AC and CLCs in the NP have similar gene expression profiles (Kamatani et al., 2022). However, we did not perform a downstream analysis, including that of the interaction between clusters. Thus, the interactions between NCs and CLCs in the NP remain unknown, and signals that contribute to the transcriptomic similarity between CLCs and chondrocytes still require elucidation.

In this study, we performed scRNA-seq analysis of the NP and AC of cynomolgus monkeys, combined these data with previously published data, and analyzed the intercellular signaling network between cell clusters in the NP and AC. In addition, we performed experiments to validate the scRNA-seq findings.

## 2 Materials and methods

### 2.1 Ethics statement

All methods were performed following the relevant guidelines and regulations. Male cynomolgus monkeys (*Macaca fascicularis*) were used in this study. All animal experiments were approved by the Institutional Animal Committee of Kyoto University (1674-17). Experiments on mice were approved by the Institutional Animal Committee of Osaka University (03-044-014).

### 2.2 Single-cell preparation

Three-year-old male cynomolgus monkeys (Ina Research, Nagano, Japan) were euthanized. In terms of age, three-year-old monkeys correspond to approximately 6-year old humans. Sample collection, cellular barcoding, and flow cytometry were performed as previously reported (Kamatani et al., 2022). Briefly, NP (L3/4) and AC (femoral condyle) were harvested from one cynomolgus monkey and AC (femoral condyle) was harvested from another. The NP samples were gelatinous, and AC samples were cut into small pieces (2 mm) using a biopsy punch. Next, both samples were washed with preparation medium (RPMI-1640 [Nacalai Tesque, Kyoto, Japan] with 0.2% fetal bovine serum [FBS] [Gibco] and 10 mM HEPES) and transferred to a 6-well plate containing digestion medium (preparation medium with 0.2 mg/mL Liberase TM [Roche, Basel, Switzerland] and 2 kU/mL DNaseⅠ [Merck, Rahway, NJ, United States]) within 2 h of sacrifice. The plate was then placed on a shaker and incubated for 3 h at 37°C and 5% CO_2_. After dissociation, the samples were applied to a 70 μm cell strainer (BD Biosciences, Franklin Lakes, NJ, United States), followed by the next step of the cell barcoding process.

For cell surface labeling, we biotinylated cell surface proteins as previously described (Sugimoto et al., 2022). Cells were resuspended in 1 mL phosphate Buffered Saline (PBS) supplemented with 1% FBS and 1 ng EZ-Link Sulfo-NHS-Biotin (Thermo Fisher Scientific, Waltham, MA, United States) for 10 min at 4°C, washed twice with PBS supplemented with 3% FBS, and then washed with Cell Staining Buffer (BioLegend, San Diego, CA, United States). Next, the cells were stained with 0.6 μg/mL of Totalseq (A0951-A0,954 [BioLegend]) for 20 min at 4°C for cellular barcoding, washed with Cell Staining Buffer, washed with PBS supplemented with 1% FBS, and resuspended in PBS supplemented with 10% FBS and 1 μM Sytox blue dead cell stain (Invitrogen, Waltham, MA, United States) for 5 min at room temperature.

Barcoded live dissociated cells (PE-positive and Sytox blue-negative) were sorted and suspended in PBS supplemented with 20% FBS. The data were collected on a FACS Aria II flow cytometer (BD Bioscience) and analyzed with BD FACS Diva 9.0.1 (BD Biosciences).

### 2.3 Library preparation, sequencing, and fastq file preprocessing

Library preparation and the first processing of paired-end fastq files were performed according to the workflow in the previously reported TAS-seq method (Shichino et al., 2022). In brief, sorted single-cell suspensions were used to synthesize cDNA with BD Rhapsody Express Single-Cell Analysis System (BD Biosciences) using a BD Rhapsody Targeted and Abseq Reagent kit (BD Biosciences) following the manufacturer’s instructions. After reverse transcription, the resultant BD Rhapsody beads were treated with exonuclease I at 37°C for 60 min at 1,200 rpm on a Thermomixer C with Thermotop. Resultant beads were immediately chilled on ice; the supernatant was removed and washed, resuspended, and stored at 4°C. During the washing step, bead-containing DNA LoBind tubes were replaced twice.

cDNAs were amplified by the TAS-seq method at Immunogeneteqs Inc. (Noda city, Chiba, Japan) and sequenced with an Illumina Novaseq 6,000 sequencer (Illumina, San Diego, CA, United States), using a Novaseq 6,000 S4 reagent kit v1.0 or v1.5.

TAS-Seq data cDNA reads were mapped to the reference genome (Macaca_fascicularis) using HISAT2-2.2.1 (Kim et al., 2019) and the following parameters: q -p 6 –rna-strandness F–very-sensitive–seed 656,565 –reorder–omit-sec-seq–mm. For the HISAT2 index build, a corresponding ensembl gtf file was filtered to retain protein-coding RNA, long non-coding RNA, and T cell chain/immunoglobulin chain annotations according to the 10X Genomics’s method (https://support.10xgenomics.com/single-cell-gene-expression/software/pipelines/latest/advanced/references#mkgtf). Then, the cell barcode information of each read was added to the HISAT2-mapped BAM files, and associated gene annotations were assigned using featureCounts v2.0.2 (Liao et al., 2014) and the following parameters: T 2 -Q 0 -s 1 -t gene -g gene_name–primary -M -O–largestOverlap–fraction -R BAM. In the featureCounts analysis, a “gene” annotation was used to capture unspliced RNA information for the RNA velocity analysis, and primary annotations were kept. The resulting BAM file was split using valid cell barcodes and nim 1.0.6 and hts-nim v0.2.23, the split files were processed into loom files using velocyto run (vesion 0.17.17) with the -c and -U options, and the loom files were concatenated using the loompy. combine function (version 3.0.6) (La Manno et al., 2018).

### 2.4 ScRNA-seq data processing and analysis

The sequencing depth was approximately 220,000 reads per cell. The loom file data were imported into the Seurat R package (version 4.0.2) (Hao et al., 2021). Spliced, unspliced, and ambiguous reads were combined. The scRNA-seq datasets of cynomolgus monkeys that we previously obtained (GSE197380) with the same method were also imported and combined. Quality control was performed on all datasets, retaining cells with 200–9,000 detected genes and < 5% mitochondrial transcripts. We performed log-normalization of all datasets, using a “scale factor” of 1,000,000 molecules for each cell, identifying the top 5,000 highly variable genes because the parameter of “scale factor” of 1,000,000 was previously reported to be suitable for the TAS-seq dataset (Shichino et al., 2021). Canonical correlation analysis was performed to correct batch effects among all samples. We calculated cell cycle phase scores from the integrated data using the CellCycleScoring function and scaled the data by regressing them to mitigate the effects of cell cycle heterogeneity. We performed principal component analysis (PCA) on the scaled expression values using the first 30 principal components (PCs) to build an SNN graph and for data clustering, dimension reduction with Uniform Manifold Approximation and Projection (UMAP), and data projection to two dimensions. We set the resolution parameter at 0.27. Marker genes were detected using the FindAllMarkers function, and differentially expressed genes (DEGs) were detected using the FindMarkers function; adjusted *p*-values less than 0.05 were considered significant. For all other parameters, the default parameters were used unless otherwise specified. In the analysis of NCs, we set the resolution parameter at 0.1 and performed re-clustering.

### 2.5 Ingenuity pathway analysis (IPA)

DEGs between clusters were identified using the FindMarkers function in Seurat. The DEGs were then subjected to IPA (QIAGEN Inc., version 81348237) (Krämer et al., 2014). For pathway analysis with the IPA tool, we defined a pathway with a z. score >2.0, and -log (*p*-value) > 1.3 as an activation pathway.

### 2.6 Cell–cell communication analysis

The integrated data analyzed by Seurat were imported into the CellChat R package (version 1.1.2) to infer cell–cell communication in all genes (Jin et al., 2021). Signaling pathways were analyzed using CellChatDB.human. For all parameters, default parameters were used to compute the communication probability and infer the cellular communication network and cell–cell communication at the signaling pathway level. Visualization of generated data was performed using a circular plot.

### 2.7 Trajectory inference

The data clustered by Seurat in the analysis of NCs were imported into the monocle3 R package (version ‘0.2.3.3’). The inferred trajectories were projected onto UMAP plots generated by Seurat. For all parameters, the default parameters were used to compute the pseudotime.

### 2.8 Histological analysis

The IVD samples from a cynomolgus monkey were dissected from the adjacent growth plates, fixed in Mildform 10 N (FUJIFILM Wako) at 4°C, dehydrated in 30% sucrose overnight at 4°C, embedded in SCEM compound (SECTION-LAB), and then cryosectioned at 5 μm according to the method described by Kawamoto (Kawamoto, 2003).

The AC samples from cynomolgus monkeys were harvested from the remnant of the femoral condyle cut with a biopsy punch for scRNA-seq, fixed in Mildform 10 N at 4°C, decalcified with K-CX (Falma), neutralized with sulfuric acid (FUJIFILM Wako), dehydrated in graded ethanol, and embedded in paraffin wax.

Fourteen-week-old rat coccygeal IVD samples were harvested, fixed with 4% paraformaldehyde (Nacalai Tesque), decalcified with 10% EDTA (Dojindo Laboratories, Kumamoto, Japan), dehydrated in graded ethanol, and embedded in paraffin.

The IVD cryosections of a cynomolgus monkey were blocked with Blocking One Histo (Nacalai Tesque) and then incubated with primary antibodies diluted in Blocking One Histo at 4°C overnight. Next, 4′,6-diamidino-2-phenylindole (DAPI) (1:1,000, Dojindo Laboratories) and secondary antibodies were incubated for 1 h at room temperature, and images were captured using BZ-9000 (Keyence, Osaka City, Osaka, Japan). The primary antibodies used were anti-Cytokeratin19 (CK19) (1:800, ab52625, Abcam, Cambridge, United Kingdom) and anti-MGP (1:800, 60,055-1-lg, Proteintech, Rosemont, IL, United States), and the secondary antibodies used were Alexa Fluor 488 (1:1,000, #A-11029, Thermo Fisher Scientific) and Alexa Fluor 546 (1:1,000, #A-11010, Thermo Fisher Scientific).

The paraffin sections of AC and rat IVD samples were deparaffinized and incubated in 1 mM EDTA buffer (pH 8.0) at 80°C for 15 min to retrieve the antigen and treated with 10 mg/mL hyaluronidase at 37°C for 40 min. Immunostaining was performed using the catalyzed signal amplification (CSA) system (CSA II Biotin-free Tyramide Signal Amplification System Kit (Agilent Technologies, Santa Clara, CA, United States)) according to the manufacturer’s protocol. A BZ-9000 was used for all the analyses. The primary antibodies used were anti-Sonic hedgehog (SHH) (1:50, sc-365112, Santa Cruz Biotechnology, Dallas, TX, United States), anti-Indian hedgehog (IHH) (1:50, ab52919, Abcam), anti-IGFBP6 (1:1,000, 67567-Ig, Proteintech), and anti-Ki67 (1:500, ab16667, Abcam) for AC samples and anti-SHH (1:200, sc-365112, Santa Cruz Biotechnology), anti-IHH (1:200, ab52919, Abcam), anti-Ki67 (1:500, ab16667, Abcam) and anti-CK19 (1:500, ab52625, Abcam) for rat IVD samples, which were incubated overnight.

### 2.9 Primary chondrocytes culture

Primary chondrocytes were harvested from the epiphyseal cartilage of the knee joints and femoral heads of newborn C57BL/6 mice, as reported previously (Gosset et al., 2008). Briefly, cartilage was digested with 3 mg/mL collagenase D (Roche) in culture medium, which was a mixture of Dulbecco’s Modified Eagle Medium (DMEM) high glucose (Sigma-Aldrich, St. Louis, MO, United States) and DMEM/nutrient mixture F-12 Ham medium (Sigma-Aldrich) supplemented with 5% FBS (Gibco) and 1% penicillin/streptomycin (Gibco). Cells (5 × 10^5^) were seeded in a 10-cm dish and cultured in a culture medium without 1% penicillin/streptomycin. After the cells were confluent, they were passaged into 24-well plates and cultured for 3–5 days. Then, 100 or 330 ng/mL recombinant SHH (rSHH) (Peprotech, Rocky Hill, NJ, United States) was added, and the cultures were incubated for 24 h under hypoxia (1% O_2_) using the BIONX-1 hypoxic culture kit (Sugiyamagen, Tokyo, Japan).

### 2.10 Western blot analysis of cultured primary chondrocytes

Cultured cells were lysed with RIPA lysis and extraction buffer (Thermo Fisher Scientific), prepared with Sample Buffer (Invitrogen) and Reducing Agent (Invitrogen), and heated at 95°C for 10 min. The lysate was applied to the 4%–12% gel (Invitrogen) to separate proteins and transferred to nitrocellulose membranes (Invitrogen). Membranes were incubated with anti-β-actin (#4967, Cell Signaling Technology, Danvers, MA, United States) and anti-hypoxia-inducible factor 1-alpha (HIF-1 alpha) antibodies (ab16066, Abcam) overnight and then with mouse anti-rabbit IgG-HRP (sc-2357, Santa Cruz Biotechnology) or m-IgG2a BP-HRP (sc-542731, Santa Cruz Biotechnology). Immunoreactive bands were visualized using SuperSignal^™^ West Dura Extended Duration Substrate (Thermo Fisher Scientific) and Fusion SOLO.7 S.EDGE (Vilber-Lourmat) and quantified with Evolution Capt (Vilber-Lourmat). HIF-1α expression was calculated as the ratio of HIF-1α expression to *β*-actin expression. The amount of HIF-1α expression in the control in each membrane was set to 1, and the rest of the samples were normalized. We compared the amount of HIF-1α expression in the rSHH treatment group to that in the control group using Prism8 (GraphPad Software, San Diego, CA, United States).

### 2.11 Statistical analysis

The Kruskal–Wallis test with post-hoc Dunn’s multiple comparison test was used. Statistical significance was set at *p* < 0.05.

## 3 Results

### 3.1 AC and NP contained cell clusters with similar gene expression profiles

**FIGURE 1:**
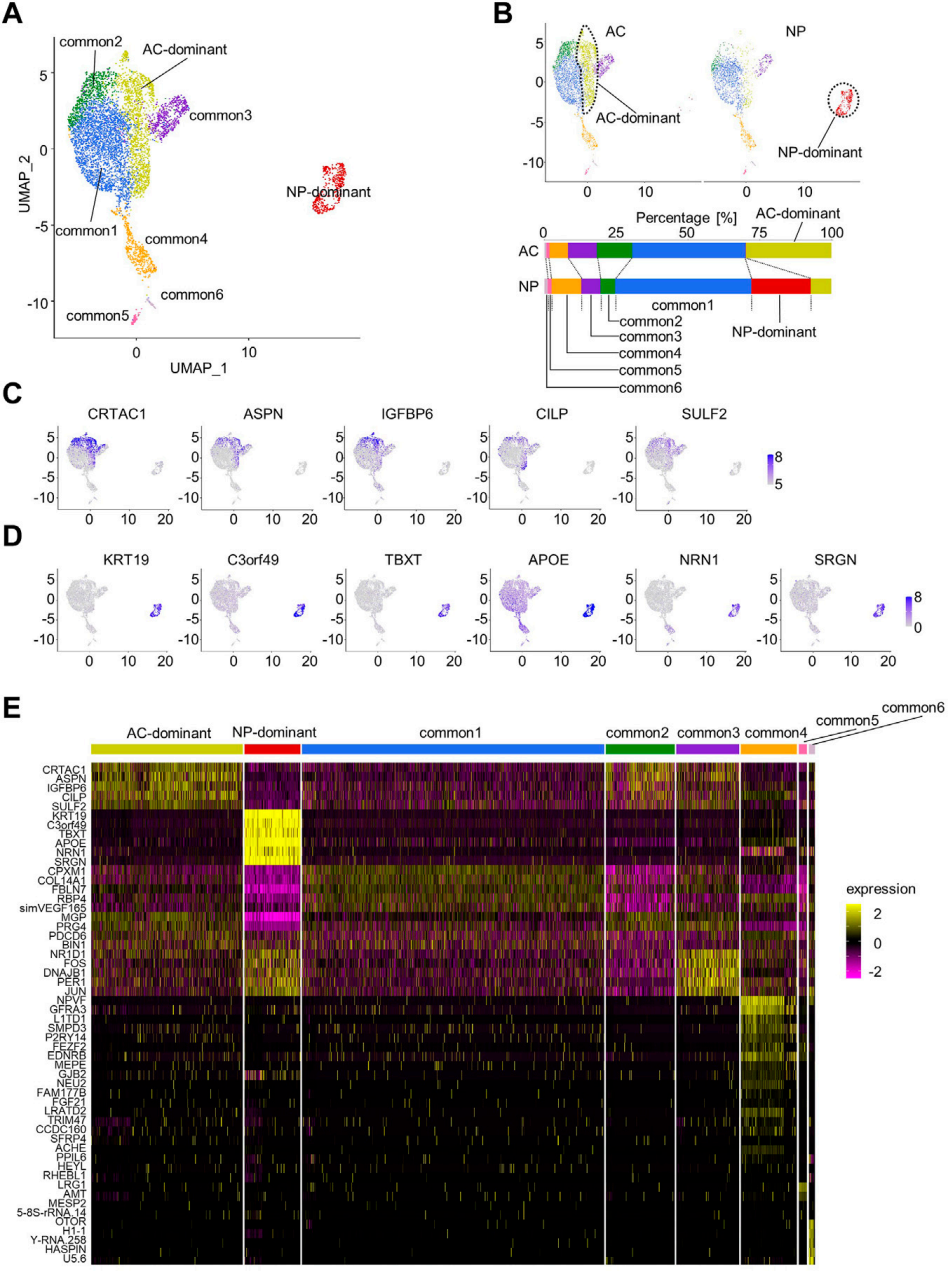
Characterization of articular cartilage (AC) and the nucleus pulposus (NP) by scRNA-seq. **(A)** Uniform Manifold Approximation and Projection (UMAP) plot for AC and the NP (total of 6,607 cells). Eight clusters were visualized by UMAP plot. **(B)** UMAP plot for AC or NP samples. The fraction of each cluster in the AC or the NP is shown. **(C)** Feature plots of marker genes for the AC-dominant cluster. **(D)** Feature plots of marker genes for the NP-dominant cluster. **(E)** Heatmap showing the scaled expression data of marker genes for each cluster.

We harvested the NP from the lumbar IVD (L3/4) and the AC from the left femoral condyle of a cynomolgus monkey (3-year-old). Additionally, we harvested the AC from the left femoral condyle of another cynomolgus monkey (3-year-old). We subjected these samples to scRNA-seq analysis (Supplementary Figure S1) and deposited the data in GEO database (GSE222449). We combined these data with scRNA-seq data previously obtained using the same method (GSE197380) (Kamatani et al., 2022) and used for analysis using Seurat (Supplementary Table S1). The total number of cells was 7,032, with an average of 220,021 reads per cell. After removing doublets and quality control, the remaining 6,607 cells were used for secondary analysis. We integrated the data for batch correction using the IntegrateData function (Stuart et al., 2019), calculated cell cycle phase scores from the data, and regressed them to mitigate the effects of cell cycle heterogeneity. We then performed PCA, data clustering, dimension reduction using UMAP, and two-dimensional projection. The resolution parameter was set to 0.27, revealing 8 cell clusters (Figure 1A). One cluster was almost exclusively composed of NP cells, and therefore, we named it the “NP-dominant cluster.” Another cluster was mainly composed of AC cells, and therefore, we named it the “AC-dominant cluster.” The other six clusters consisted of both NP cells and AC cells, and were therefore named “common clusters” (common1–6 clusters; Figures 1A, B, Supplementary Figure S2). The existence of common clusters indicated that the AC and NP contained cells with similar gene expression profiles.

Next, marker genes for AC- and NP-dominant clusters were examined. Candidate marker genes were detected using the FindAllMarkers function, and those with an adjusted *p*-value < 0.05 were identified as marker genes and sorted by adjusted *p*-value (Supplementary Table S2). Marker genes for the AC-dominant cluster included *CRTAC1*, *ASPN*, *IGFBP6*, *CILP*, and *SULF2*. The feature plot function confirmed that these genes were preferentially expressed in the AC-dominant cluster (Figures 1C, E, Supplementary Table S2). Marker genes for NP-dominant clusters included *KRT19, TBXT, C3orf49*, *APOE*, *NRN1,* and *SRGN*, which were previously reported as markers for NCs (Tessier and Risbud, 2021; Kamatani et al., 2022). The feature plot function confirmed that these genes were preferentially expressed in the NP-dominant cluster (Figures 1D, E, Supplementary Table S2).

To confirm reproducibility, we reanalyzed scRNA-seq data from each monkey. NP-dominant, AC-dominant, and common clusters of cells were identified, and were shown to express respective marker genes in each monkey (Supplementary Figures S3–S5), suggesting reproducibility.

### 3.2 Characteristics of each cluster

The NP-dominant cluster exclusively expressed *KRT19* and *TBXT*, previously reported as markers for NCs (Mohanty et al., 2019; Tessier and Risbud, 2021), indicating that cells in the NP-dominant cluster in the NP are NCs. This finding suggests that the remaining cells in common clusters one to six in the NP corresponded to CLCs.

To characterize common and AC-dominant clusters, we compared their marker genes with those of the chondrocyte clusters in human AC reported by Ji et al. (2019). Articular chondrocytes were divided into six clusters: homeostatic chondrocytes (HomCs), regulatory chondrocytes (RegCs), cartilage progenitor cells (CPCs), prehypertrophic chondrocytes (preHTCs), hypertrophic chondrocytes (HTCs), and fibrocartilage chondrocytes (FCs).

**FIGURE 2:**
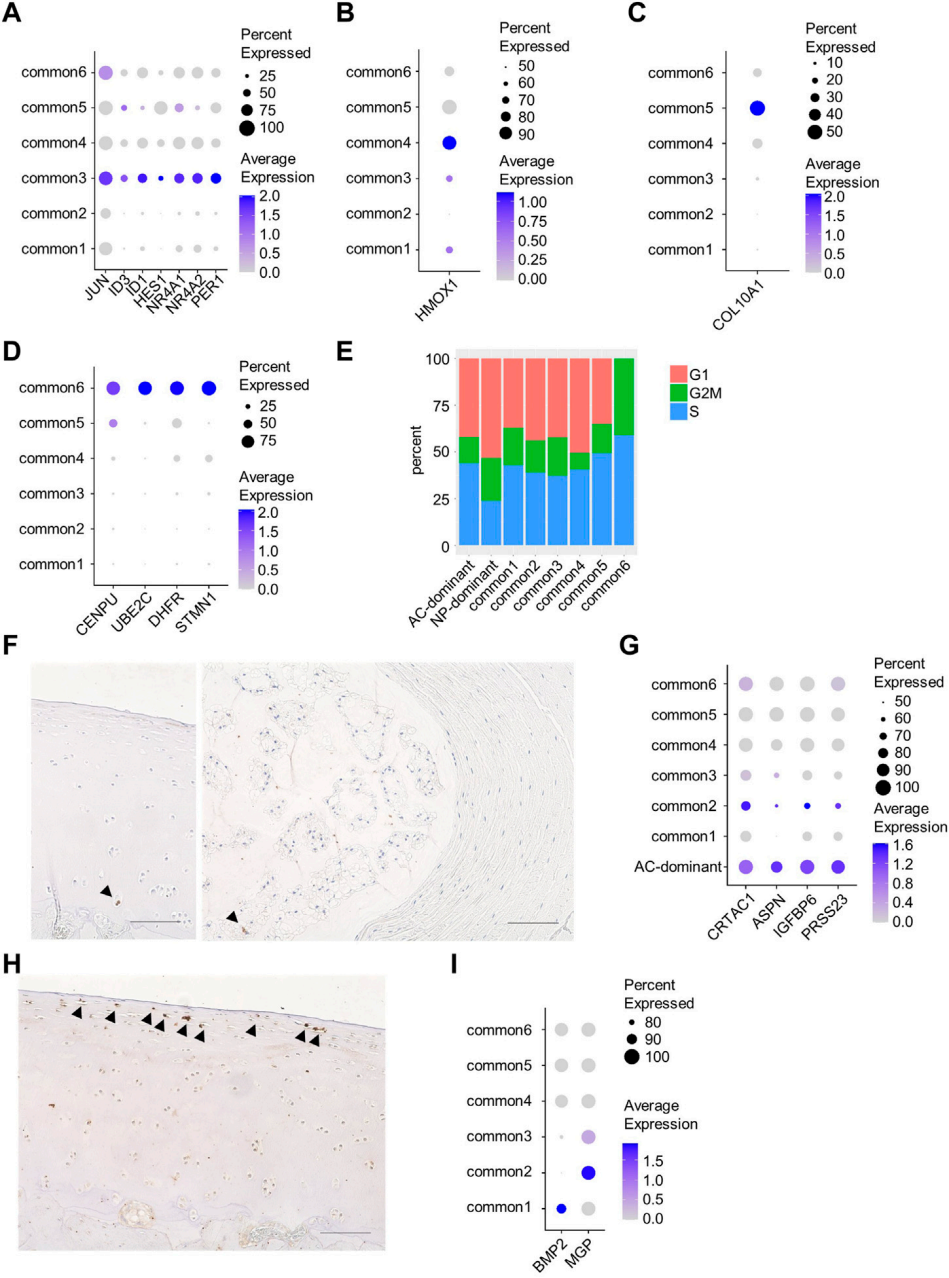
Characterization of each cluster. **(A)** Dot plots of characteristic genes of the common3 cluster. **(B)** Dot plots of characteristic genes of the common4 cluster. **(C)** Dot plots of characteristic genes of the common5 cluster. **(D)** Dot plots of characteristic genes of the common6 cluster. **(E)** The fraction of cell cycle phases in each cluster. **(F)** Immunostaining of sections from monkey AC (left) and rat NP (right) using anti-Ki67 antibody. *Arrowheads* indicate cells expressing Ki67. **(G)** Dot plots of marker genes for preHTCs or FC. **(H)** Immunostaining of sections from monkey AC using anti-IGFBP6 antibody. *Arrowheads* indicate cells expressing IGFBP6. **(I)** Dot plots of characteristic genes of the common1 or 2 cluster. Scale bars, 100 µm.

The common3 cluster corresponded to HomCs because they shared *JUN*, *ID3*, *ID1*, *HES1*, *NR4A1*, *NR4A2*, and *PER1* as marker genes (Figure 2A, Supplementary Table S2). The common4 cluster corresponded to RegCs because *HMOX1*, a marker gene for RegCs, was identified as a marker for the common4 cluster (Figure 2B, Supplementary Table S2). The common5 cluster corresponded to HTCs as both clusters preferentially expressed *COL10A1* (Figure 2C). The common6 cluster potentially corresponded to the CPC cluster because it showed high proliferative activity, as indicated by high G2M and S scores, and preferentially expressed *CENPU*, *UBE2C*, *DHFR*, and *STMN1*, marker genes for CPCs (Figures 2D, E, Supplementary Table S2). Immunohistochemical analysis revealed a few cells expressing Ki67 in AC and NP (Figure 2F). The marker genes for the AC-dominant cluster included *CRTAC1* and *ASPN*, which are also the marker genes for preHTCs, and *IGFBP6* and *PRSS23*, which are also the marker genes for FCs. This suggested that the AC-dominant cluster was a cell population composed of preHTCs and FCs (Figure 2G, Supplementary Table S2). Immunohistochemical analysis revealed cells expressing IGFBP6 in the superficial zone of AC (Figure 2H), suggesting that AC-dominant cells correspond to superficial zone cells. The common1 and common2 clusters corresponded to none of the chondrocyte clusters proposed by Ji et al. (2019). Notably, cells in the common1 cluster preferentially expressed *BMP2*, and those in the common2 cluster preferentially expressed *MGP* (Figure 2I, Supplementary Table S2).

### 3.3 Common clusters were enriched for *GLI1* as an upstream regulator

**FIGURE 3:**
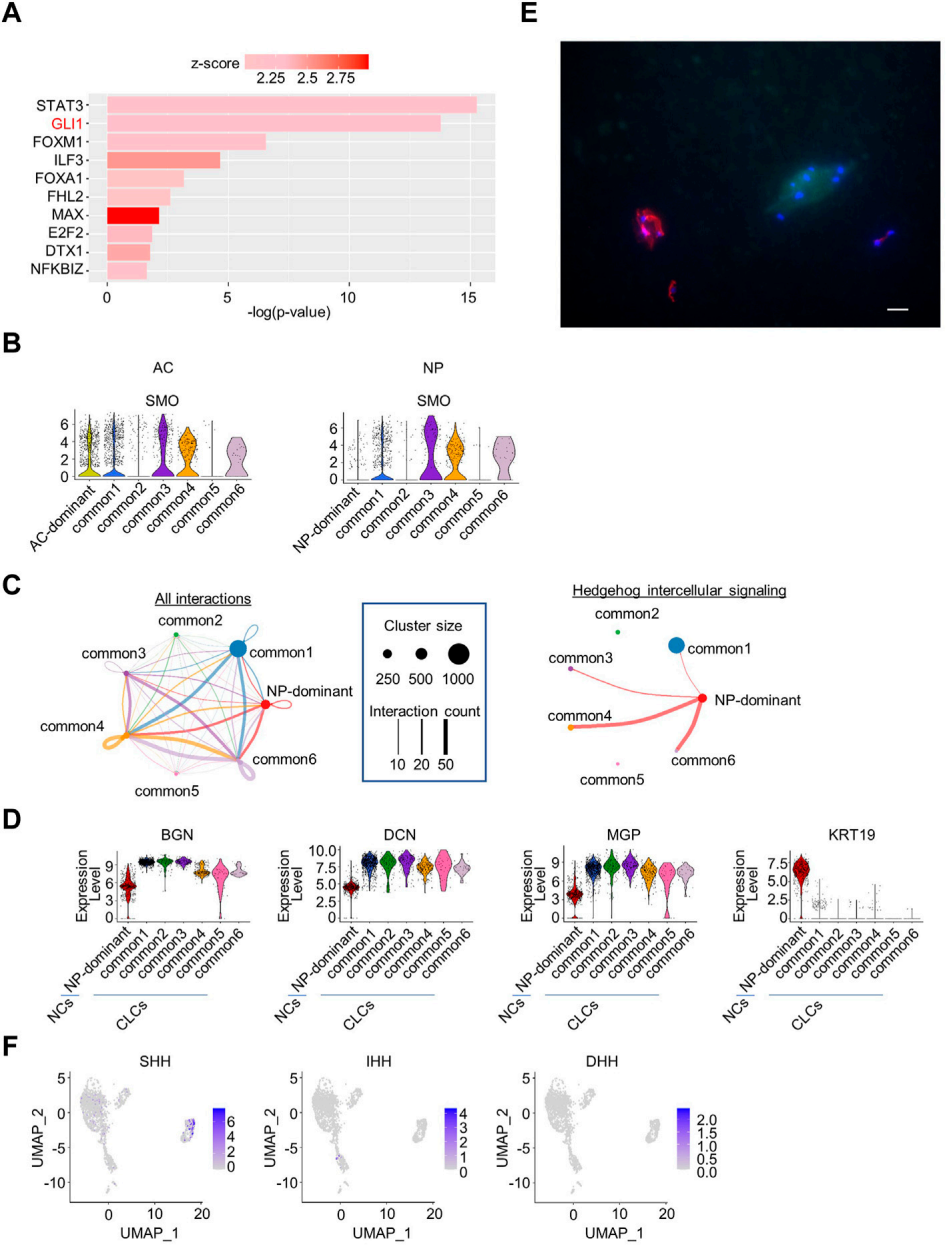
Upstream regulator analysis of NP and AC samples combined, and cell–cell interaction analysis of NP samples. **(A)** Transcription regulators enriched in common clusters in the analysis of NP and AC samples combined. **(B)** Violin plots showing the expression levels of *SMO* in the AC or NP. **(C)** Intercellular signaling between clusters identified by CellChat analysis in the NP. *Left*, circle plot of cell–cell interactions is indicated. Each cluster is indicated by a dot. The size of the dot represents the number of cells constituting the cluster, and the number of interactions is represented by the thickness of the line. *Right*, circle plot of hedgehog intercellular signaling. Hedgehog signaling was sent exclusively from NP-dominant clusters to common1, 3, 4, and 6 clusters. **(D)** Violin plots of marker genes for chondrocyte-like NP cells (CLCs) (*BGN*, *DCN,* and *MGP*) and notochordal NP cells (NCs) (KRT19) in the NP samples. **(E)** Immunostaining of a cynomolgus monkey intervertebral disc (IVD) (anti-cytokeratin (CK)19 antibody (red), 4′,6-diamidino-2-phenylindole (DAPI) (blue), and anti-matrix gla protein (MGP) antibody (green). Scale bar: 20 μm. **(F)** Feature plot showing the expression of hedgehog family members in NP samples.

To search for signaling molecules that control the six common clusters, DEGs between the common clusters and the other clusters (AC- and NP-dominant clusters) were identified and subjected to QIAGEN IPA (Krämer et al., 2014). The common clusters were enriched for genes related to 10 transcriptional factors, including *GLI1* (Figure 3A), the nuclear mediator of the hedgehog pathway (Lee et al., 1997).

To confirm that hedgehog pathways are activated in cells in common clusters in both the NP and AC, we analyzed the expression of the SMO protein, which mediates hedgehog signaling. The expression of *SMO* was elevated in some common clusters in both the NP and AC (Figure 3B), supporting the hypothesis that hedgehog pathways are activated in some common clusters in both the AC and NP.

### 3.4 Hedgehog signaling from some NCs to CLCs was inferred as intercellular signaling in NP

Next, we analyzed cell–cell interactions between clusters in the NP and AC. CellChat analysis of NP cells revealed 27 intra- and inter-cluster signals, including hedgehog signals. Interestingly, the hedgehog signals were exclusively sent from the NP-dominant cluster (Figure 3C, Supplementary Figure S6). The expression of marker genes for NCs suggested that the NP-dominant cluster cells corresponded to NCs (Figure 3D). To investigate whether the common cluster cells in the NP corresponded to CLCs, we analyzed the expression of markers in NP cells. The common clusters highly expressed genes such as *BGN* and *DCN* (Figure 3D)*,* which are upregulated in CLCs (Chen et al., 2006), suggesting that the common cluster cells corresponded to CLCs. To confirm the reproducibility across individual monkeys, we reanalyzed scRNA-seq data for each monkey. The violin plot indicated that NP-dominant cluster cells highly expressed KRT19 and that common cluster cells highly expressed matrix gla protein (MGP) in all individual monkeys, confirming reproducibility (Supplementary Figure S7). To further confirm that the NP was histologically composed of NCs and CLCs, we prepared frozen sections of IVDs from a cynomolgus monkey and performed fluorescent immunostaining with antibodies against CK19 (KRT19), a marker for NCs, and MGP. *MGP* was preferentially expressed in the common clusters but not in NP-dominant clusters (Figure 3D) and is reported to be expressed in the NP in aged individuals (Rutges et al., 2010). The results indicate that the NP contained 2 cell types, with exclusive expression of either CK19 or MGP (Figure 3E). These results collectively suggested that hedgehog signaling is transmitted from NCs to CLCs. To determine which of the three hedgehog family members (Sonic, Indian, and Desert) was sent from NCs as a ligand, we examined the expression of each hedgehog ligand in scRNA-seq data and found that only *SHH* was preferentially expressed in a part of NCs (Figure 3F).

In contrast, CellChat analysis in the AC detected 25 interactions between clusters, which did not include hedgehog signaling (Supplementary Figure S8). None of the hedgehog ligands was expressed in AC cells in the scRNA-seq data (Supplementary Figure S9). These results suggested that the hedgehog pathway in common cluster cells in the AC is activated by ligands of unknown origin.

### 3.5 Hedgehog proteins were secreted from NCs in the NP and subchondral bone in the AC

**FIGURE 4:**
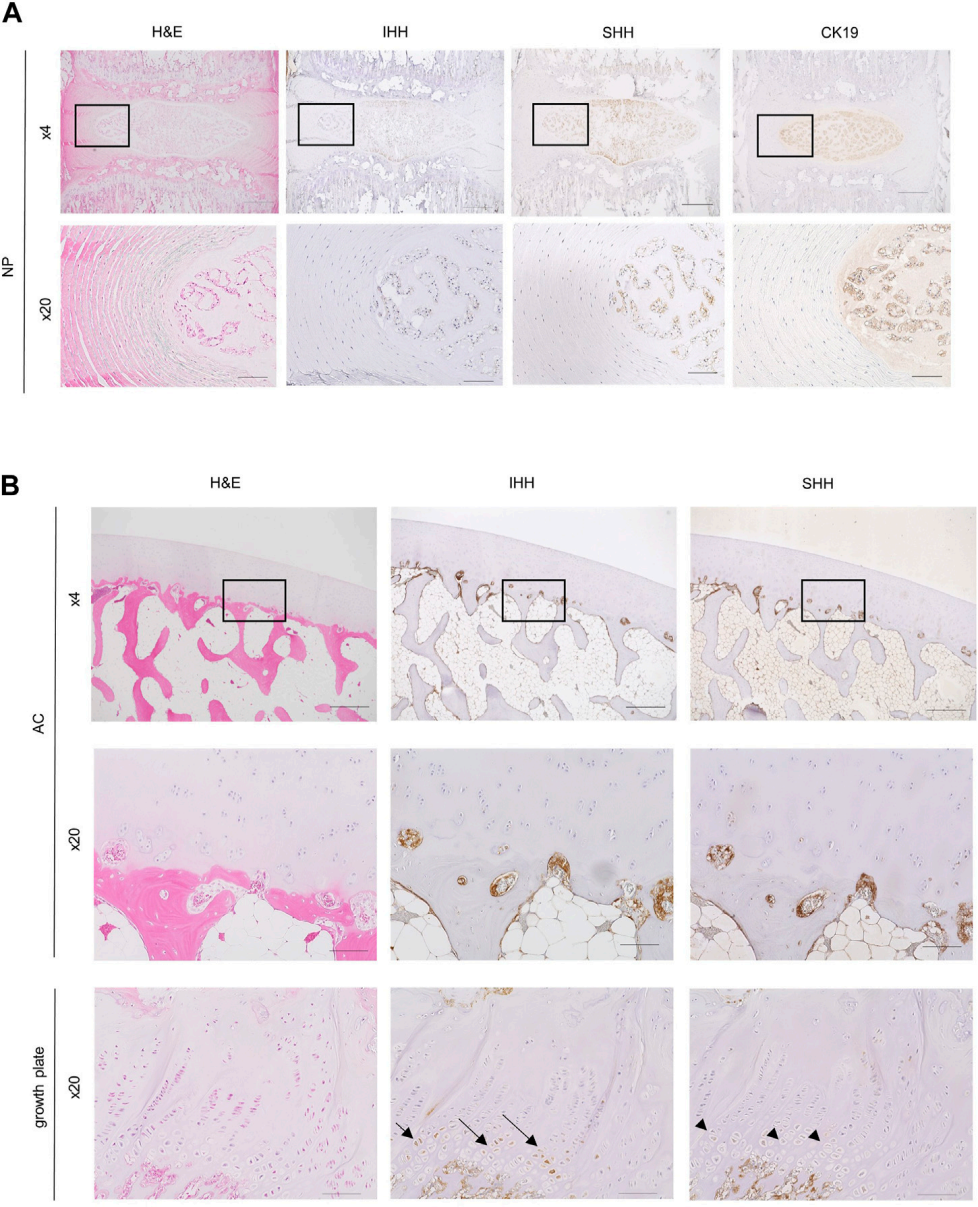
Histological and immunohistochemical analysis of sections of the IVD and AC. **(A)** Rat IVD histological sections were stained with hematoxylin-eosin (*left column*) and immunostained with anti-Indian Hedgehog (IHH) antibody (*second column*), anti-sonic hedgehog (SHH) antibody (*third column*), and anti-CK19 antibody (*right column*). A magnified view of the boxed region in the top row is shown in the bottom row. Scale bars: top row, 500 μm; bottom row, 100 μm. **(B)** Histological sections of monkey AC (*top* and *middle rows*) and growth plate cartilage (*bottom row*) stained with hematoxylin-eosin (*left*), anti-IHH antibody (*middle*), and anti-SHH antibody (*right*). Magnified images of the boxed regions at the *top* are shown in the *middle*. Note that prehypertrophic chondrocytes in the growth plate cartilage used as controls were recognized by the anti-IHH antibody (arrows) but not by the anti-SHH antibody (arrowheads). Bone marrow cells attached to the bone were recognized by both anti-IHH and anti-SHH antibodies. Scale bars: top row, 500 μm; middle and bottom rows, 100 μm.

Since scRNA-seq analysis indicated that *SHH* was expressed in some NCs in the NP, we examined whether it was also expressed at the protein level in NCs using rat caudal IVDs. Immunostaining showed that SHH was expressed in some of the NCs with vacuoles (Figure 4A). IHH expression was not detectable. This result supported the idea that NCs supply SHH proteins as ligands for hedgehog signaling to CLCs.

Next, to identify the source of hedgehog ligands in the AC, we performed immunohistochemical staining of paraffin sections of the distal end of femur from cynomolgus monkey. SHH and IHH were expressed in bone marrow cells that were attached to the subchondral bone (Figure 4B). These hedgehog-positive bone marrow cells were excluded from our scRNA-seq analysis because we did not include the subchondral bone when preparing single cells from the AC for scRNA-seq analysis. Similar to the results of our study, a previous report also indicated SHH expression in hematopoietic cells, endothelial cells, and mesenchymal stem cells in the bone marrow of the AC (Newton et al., 2019). Overall, these findings suggested that that bone marrow cells surrounding the subchondral bone supply the ligands for hedgehog signaling in the AC.

### 3.6 Hedgehog signaling potentially activates the HIF-1α pathway in articular chondrocytes and CLCs

**FIGURE 5:**
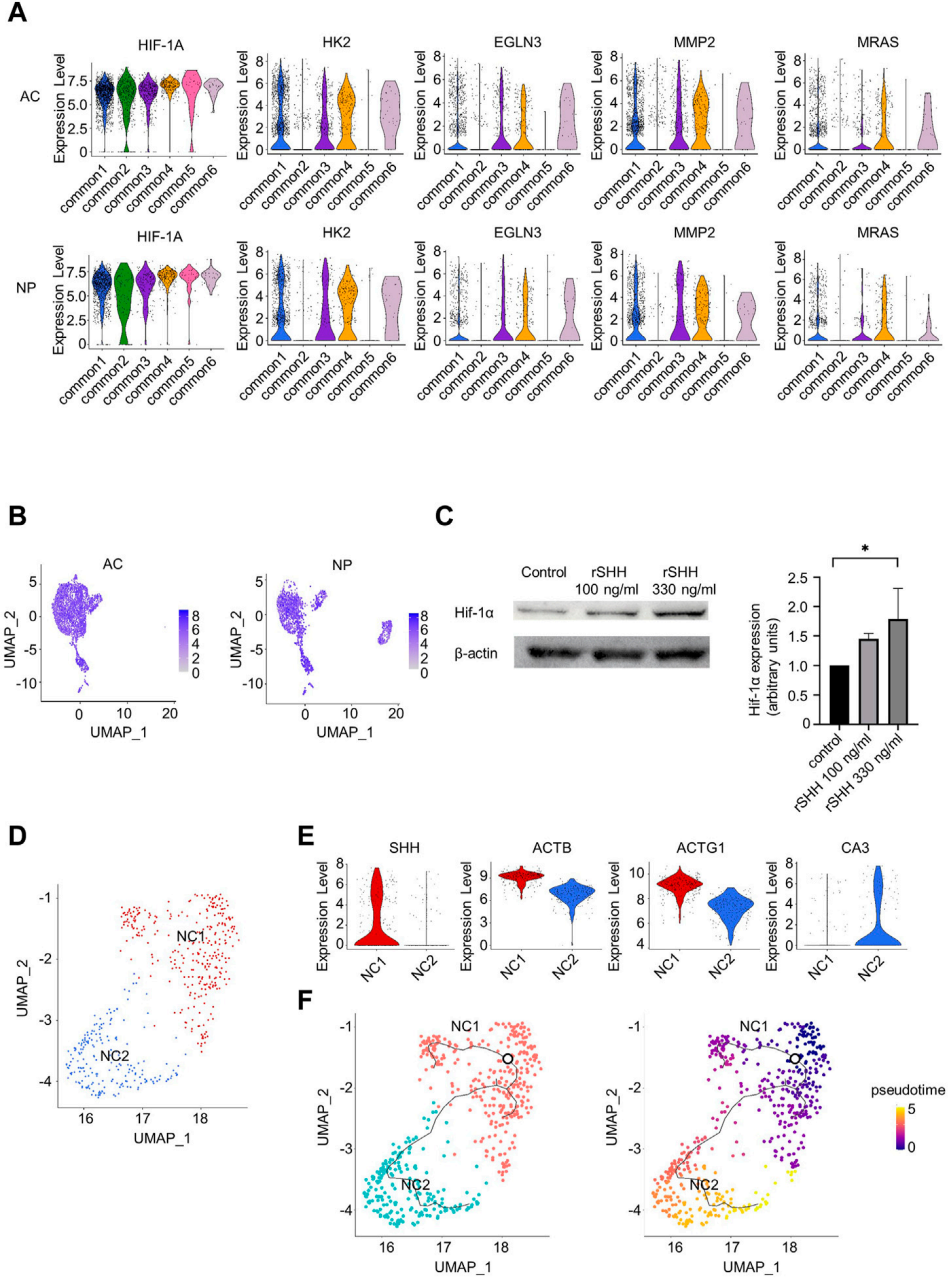
Relationship between hedgehog signaling and hypoxia-inducible factor 1-alpha (HIF-1α) signaling and re-clustering analysis of NCs. **(A)** Violine plots showing the expression levels of *HIF-1A* and HIF-1α targeting genes in the AC or NP. **(B)** Feature plots of *HIF-1A*. **(C)** Immunoblot expression analysis of Hif-1α in primary chondrocytes treated with 100 or 330 ng/mL rSHH for 24 h. *Left*: blots representative of four independent experiments are shown. *Right*: quantification of Hif-1α to *β*-actin ratio. *p-value < 0.05 by Kruskal–Wallis test with *post hoc* Dunn’s multiple comparisons test (*n* = 4). **(D)** UMAP plot of NP-dominant cluster cells after re-clustering. **(E)** Violin plots showing marker genes in the NC1 or NC2 cluster. **(F)** Results of trajectory analysis by Monocle 3. *Left*, trajectories are projected onto UMAP plot of NP-dominant cluster cells. The root node, which represents the beginning of the biological process, is indicated by a circle. *Right*, pseudotime for NP-dominant cluster cells.

Hedgehog pathways appeared to be activated in some common clusters. *SMO,* which encodes a protein mediating hedgehog signaling, was highly expressed in clusters common 1, 3, 4, and 6, but not in clusters common 2 and 5 in the AC and NP, respectively (Figure 3B). Consistently, Cell Chat analysis detected hedgehog signaling from NP-dominant clusters to clusters common 1, 3, 4, and 6, but not to clusters common 2 and 5 (Figure 3C, *right*). To investigate the effect of hedgehog signaling, we identified DEGs between common clusters that appeared to receive hedgehog signaling (common 1, 3, 4, and 6) and those that did not (common 2 and 5) and subjected them to IPA in the AC and NP samples independently. We found 58 and 60 pathways significantly enriched in the AC and NP samples, respectively, with 24 pathways enriched in both samples. We were interested in the HIF-1α pathway among the 24 pathways (Supplementary Figure S10) because both the AC and NP are known to be under hypoxic conditions wherein the HIF-1α pathway is activated. The violin plot function confirmed that HIF-1α target genes were preferentially expressed in common 1, 3, 4, and 6 than in common 2 and 5 clusters in both the AC and NP (Figure 5A). The expression level of *HIF-1A* mRNA was not different between clusters (Figure 5A, B), consistent with the findings that HIF-1α activity is regulated at the protein level (Aragonés et al., 2009).

We further analyzed the relationship between hedgehog signaling and HIF-1α expression. The addition of SHH increased HIF-1α protein levels in mouse primary chondrocytes (Figure 5C), indicating that hedgehog signaling activates the HIF-1α pathway.

### 3.7 IPA revealed enrichment of TGF-β1 signaling in NCs with high *SHH* expression

As indicated by the FeaturePlot function in scRNA-seq analysis, only a part of the NCs preferentially expressed *SHH* (Figure 3F), suggesting heterogeneity within NCs. To further analyze NCs, NP-dominant cluster cells were re-clustered with the resolution parameter set to 0.1, resulting in identification of two clusters: NC1 and NC2 (Figure 5D). Interestingly, the NC1 cluster preferentially expressed *SHH*, whereas NC2 did not (Figure 5E). Consistently, immunohistochemical analysis indicated that all NCs with vacuoles expressed CK19, while part of NCs with vacuoles expressed SHH (Figure 4A). Furthermore, *ACTB* and *ACTG1* were preferentially expressed in NC1 than in NC2, whereas *CA3*, a gene expressed in NCs in individuals aged 13 years (Gan et al., 2021), was preferentially expressed in NC2 than in NC1. IPA of DEGs between NC1 and NC2 revealed that the NC1 cluster was enriched for genes related to TGF-β1 as an upstream regulator (Supplementary Figure S11). Together with a previous report that TGF-β1 increases SHH expression at the mRNA level in fibroblasts (Wen et al., 2022), this result suggests that TGF-β1 signals are involved in increasing the expression of SHH in NCs. To investigate the differential trajectories among the NCs, we next performed trajectory inference using Monocle 3 (Figure 5F, *left*). NC1 cells were predicted to be root cells that differentiated into NC2 cells by the computed pseudotime (Figure 5F, *right*).

## 4 Discussion

**FIGURE 6:**
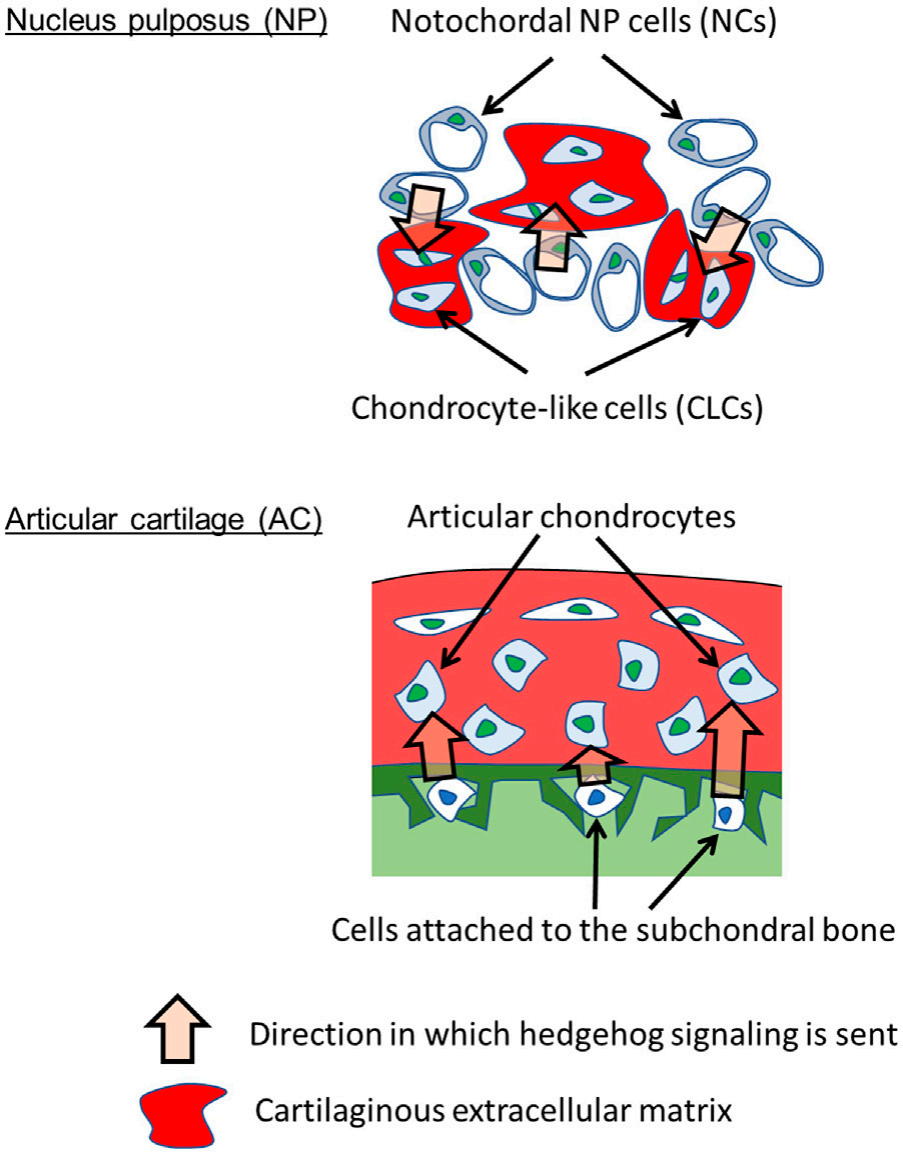
Parallel paracrine-regulation by hedgehog in NP and AC. The results suggest that hedgehog signaling is sent from NCs to CLCs in NP and from subchondral bone cells to articular chondrocytes in AC. Hedgehog signaling activates HIF-1α signaling.

The importance of hedgehog pathways in NP and AC homeostasis has been reported. Modulation of hedgehog pathways causes osteoarthritis (Lin et al., 2009) and IVD degeneration in mice (Bao et al., 2021). Gli1 has been found to be expressed in NP (Li et al., 2019). However, the underlying mechanisms by which the NP and AC acquire hedgehog pathways in parallel remain to be elucidated. During development, the notochord is formed at embryonic days E) 9–11 in mice (McCann and Seguin, 2016) and Carnegie stages 8–12 (E 15–30) in humans (de Bree et al., 2018). The notochord expresses and secretes SHH to induce the differentiation of surrounding cells, including mesenchymal cells that form vertebral bodies (Choi et al., 2012). Notochord cells become NCs in the NP after birth and continue to express SHH (Dahia et al., 2009). Our study suggested that SHH from NCs activates the hedgehog pathway in CLCs (Figure 6, *top*). Although the precise mechanism by which SHH regulates IVD degeneration is unknown, our results suggest that the hedgehog pathway in CLCs underlies IVD homeostasis. During limb development, IHH is expressed in the prehypertrophic chondrocytes of the primordial cartilage. IHH stimulates the proliferation of proliferative chondrocytes (St-Jacques et al., 1999). In addition, IHH induces the expression of PTHrP, which inhibits chondrocyte hypertrophy and regulates the height of the proliferative chondrocyte zone (Vortkamp et al., 1996). Although the role of hedgehog pathways and the expression of hedgehog proteins are less understood after birth than in embryos, they are essential for the homeostasis of articular chondrocytes (Lin et al., 2009). Our results suggested that bone marrow cells that attach to subchondral bone provide hedgehog ligands, which then activate the hedgehog pathway in articular chondrocytes (Figure 6, *bottom*). Although the developmental narrative of establishing hedgehog pathways differs between the NP and AC, it is interesting to assume that the parallel activation of the hedgehog pathway in the NP and AC accounts for the similarity in the transcriptional profiles between CLCs and articular chondrocytes.

The relationship between hedgehog signaling and HIF-1α has been reported. Specifically, HIF-1α regulates hedgehog pathways in tumor cells, whereas hedgehog pathways regulate HIF-1α activity in hepatic stellate cells (Zhang et al., 2017; Bhuria et al., 2019). In the current study, we found that the hedgehog pathway regulates HIF-1α activity in chondrocytes. However, it remains to be determined how the hedgehog–HIF-1α axis contributes to the homeostasis of the NP and AC.

The fact that the number of NCs decreases with age and becomes obliterated by the age of 10 poses limited opportunity to study NCs in humans. Previous studies have analyzed IVD in individuals aged 10 years or older (Gan et al., 2021), showing that the relative number of NCs to that of CLCs is low. Here, we used 3-year-old monkeys and obtained a substantial number of NCs, allowing us to conduct detailed analysis and identify hedgehog signaling from NCs to CLCs. In addition, we detected 2 cell types in NCs: one that highly expresses *SHH* (*SHH* ^*+*^ cell) and the other that does not express *SHH* (*SHH* ^*−*^ cell). *SHH* ^*−*^ cells have been previously identified in individuals aged 10 years or older (Gan et al., 2021). To the best of our knowledge, these are the first *SHH* ^*+*^ cells to be identified using scRNA-seq. Our results from trajectory analysis suggested that *SHH* ^*+*^ cells turn to *SHH* ^*−*^ cells, suggesting that NCs may lose *SHH* expression as they age, followed by their obliteration in the NP.

The limitations of this study include the difficulty of preparing single cells by enzymatic digestion of tissues. The gelatinous NP provided single cells by enzymatic digestion for 3 h, whereas it took 4 h to extract single cells from solid AC. In addition, during cell preparation, cells were in PBS supplemented with 10%–20% FBS under normoxic conditions, which may have affected transcription. scRNA-seq data indicated that the cells analyzed highly expressed genes specific to chondrocytes and NCs, suggesting that cell type properties were maintained during the process of cell preparation for scRNA-seq. However, we need to consider the possibility that the transcription profile might be altered during enzymatic digestion to extract cells from tissues when we interpret the data to infer events *in vivo*. Another limitation is that the scRNA-seq data in this study were obtained from three male monkeys. A potential difference based on sex remains to be analyzed.

Overall, our findings revealed that CLCs in the NP and chondrocytes in the AC are paracrine-regulated by hedgehog proteins from NCs and the subchondral bone, respectively (Figure 6). Hedgehog proteins promote the HIF-1α pathway in CLCs and chondrocytes, likely contributing to the transcriptomic similarity between these cells. These findings would contribute to our understanding of the mechanisms that regulate the homeostasis of the NP and AC.

## Data Availability

The datasets presented in this study can be found in online repositories. The names of the repository/repositories and accession number(s) can be found below: NCBI GEO GSE222449.

## References

[B1] AragonéS J. Fraisl P. Baes M. Carmeliet P. (2009). Oxygen sensors at the crossroad of metabolism. Cell Metab. 9, 11–22. 10.1016/j.cmet.2008.10.001 19117543

[B2] Bao J. Qian Z. Liu L. Hong X. Che H. Wu X. (2021). Pharmacological disruption of phosphorylated eukaryotic initiation factor-2α/activating transcription factor 4/Indian hedgehog protects intervertebral disc degeneration via reducing the reactive oxygen species and apoptosis of nucleus pulposus cells. Front. Cell Dev. Biol. 9, 675486. 10.3389/fcell.2021.675486 34164397 PMC8215438

[B3] Bhuria V. Xing J. Scholta T. Bui K. C. Nguyen M. L. T. Malek N. P. (2019). Hypoxia induced Sonic Hedgehog signaling regulates cancer stemness, epithelial-to-mesenchymal transition and invasion in cholangiocarcinoma. Exp. Cell Res. 385, 111671. 10.1016/j.yexcr.2019.111671 31634481

[B4] Buckwalter J. A. Mankin H. J. (1998). Articular cartilage: Tissue design and chondrocyte-matrix interactions. Instr. Course Lect. 47, 477–486. 9571449

[B5] Butler A. Hoffman P. Smibert P. Papalexi E. Satija R. (2018). Integrating single-cell transcriptomic data across different conditions, technologies, and species. Nat. Biotechnol. 36, 411–420. 10.1038/nbt.4096 29608179 PMC6700744

[B6] Chen J. Yan W. Setton L. A. (2006). Molecular phenotypes of notochordal cells purified from immature nucleus pulposus. Eur. Spine J. 15 (3), S303–S311. 10.1007/s00586-006-0088-x 16547755 PMC2335373

[B7] Chen S. Fu P. Wu H. Pei M. (2017). Meniscus, articular cartilage and nucleus pulposus: A comparative review of cartilage-like tissues in anatomy, development and function. Cell tissue Res. 370, 53–70. 10.1007/s00441-017-2613-0 28413859 PMC5645221

[B8] Choi K. S. Cohn M. J. Harfe B. D. (2008). Identification of nucleus pulposus precursor cells and notochordal remnants in the mouse: Implications for disk degeneration and chordoma formation. Dev. Dyn. 237, 3953–3958. 10.1002/dvdy.21805 19035356 PMC2646501

[B9] Choi K. S. Lee C. Harfe B. D. (2012). Sonic hedgehog in the notochord is sufficient for patterning of the intervertebral discs. Mech. Dev. 129, 255–262. 10.1016/j.mod.2012.07.003 22841806 PMC3478436

[B10] Dahia C. L. Mahoney E. J. Durrani A. A. Wylie C. (2009). Intercellular signaling pathways active during intervertebral disc growth, differentiation, and aging. Spine (Phila Pa 1976) 34, 456–462. 10.1097/BRS.0b013e3181913e98 19212276

[B11] de Bree K. de Bakker B. S. Oostra R. J. (2018). The development of the human notochord. PLoS One 13, e0205752. 10.1371/journal.pone.0205752 30346967 PMC6197658

[B12] Eyre D. R. Muir H. (1977). Quantitative analysis of types I and II collagens in human intervertebral discs at various ages. Biochim. Biophys. Acta 492, 29–42. 10.1016/0005-2795(77)90211-2 577186

[B13] Gan Y. He J. Zhu J. Xu Z. Wang Z. Yan J. (2021). Spatially defined single-cell transcriptional profiling characterizes diverse chondrocyte subtypes and nucleus pulposus progenitors in human intervertebral discs. Bone Res. 9, 37. 10.1038/s41413-021-00163-z 34400611 PMC8368097

[B14] Gosset M. Berenbaum F. Thirion S. Jacques C. (2008). Primary culture and phenotyping of murine chondrocytes. Nat. Protoc. 3, 1253–1260. 10.1038/nprot.2008.95 18714293

[B15] Hao Y. Hao S. Andersen-Nissen E. Mauck W. M. Zheng S. Butler A. (2021). Integrated analysis of multimodal single-cell data. Cell 184, 3573–3587.e29. 10.1016/j.cell.2021.04.048 34062119 PMC8238499

[B16] HeinegåRD D. Oldberg A. (1989). Structure and biology of cartilage and bone matrix noncollagenous macromolecules. Faseb J. 3, 2042–2051. 10.1096/fasebj.3.9.2663581 2663581

[B17] Ji Q. Zheng Y. Zhang G. Hu Y. Fan X. Hou Y. (2019). Single-cell RNA-seq analysis reveals the progression of human osteoarthritis. Ann. Rheum. Dis. 78, 100–110. 10.1136/annrheumdis-2017-212863 30026257 PMC6317448

[B18] Jin S. Guerrero-Juarez C. F. Zhang L. Chang I. Ramos R. Kuan C. H. (2021). Inference and analysis of cell-cell communication using CellChat. Nat. Commun. 12, 1088. 10.1038/s41467-021-21246-9 33597522 PMC7889871

[B19] Kamatani T. Hagizawa H. Yarimitsu S. Morioka M. Koyamatsu S. Sugimoto M. (2022). Human iPS cell-derived cartilaginous tissue spatially and functionally replaces nucleus pulposus. Biomaterials 284, 121491. 10.1016/j.biomaterials.2022.121491 35395453

[B20] Kawamoto T. (2003). Use of a new adhesive film for the preparation of multi-purpose fresh-frozen sections from hard tissues, whole-animals, insects and plants. Arch. Histol. Cytol. 66, 123–143. 10.1679/aohc.66.123 12846553

[B21] Kim D. Paggi J. M. Park C. Bennett C. Salzberg S. L. (2019). Graph-based genome alignment and genotyping with HISAT2 and HISAT-genotype. Nat. Biotechnol. 37, 907–915. 10.1038/s41587-019-0201-4 31375807 PMC7605509

[B22] KräMER A. Green J. Pollard J. Tugendreich S. (2014). Causal analysis approaches in ingenuity pathway analysis. Bioinformatics 30, 523–530. 10.1093/bioinformatics/btt703 24336805 PMC3928520

[B23] La Manno G. Soldatov R. Zeisel A. Braun E. Hochgerner H. Petukhov V. (2018). RNA velocity of single cells. Nature 560, 494–498. 10.1038/s41586-018-0414-6 30089906 PMC6130801

[B24] Lee J. Platt K. A. Censullo P. Ruiz I Altaba A. (1997). Gli1 is a target of Sonic hedgehog that induces ventral neural tube development. Development 124, 2537–2552. 10.1242/dev.124.13.2537 9216996

[B25] Li K. Kapper D. Youngs B. Kocsis V. Mondal S. Kraus P. (2019). Potential biomarkers of the mature intervertebral disc identified at the single cell level. J. Anat. 234, 16–32. 10.1111/joa.12904 30450595 PMC6284444

[B26] Liao Y. Smyth G. K. Shi W. (2014). featureCounts: an efficient general purpose program for assigning sequence reads to genomic features. Bioinformatics 30, 923–930. 10.1093/bioinformatics/btt656 24227677

[B27] Lin A. C. Seeto B. L. Bartoszko J. M. Khoury M. A. Whetstone H. Ho L. (2009). Modulating hedgehog signaling can attenuate the severity of osteoarthritis. Nat. Med. 15, 1421–1425. 10.1038/nm.2055 19915594

[B28] Mccann M. R. Seguin C. A. (2016). Notochord cells in intervertebral disc development and degeneration. J. Dev. Biol. 4, 3. 10.3390/jdb4010003 27252900 PMC4885739

[B29] Mccann M. R. Tamplin O. J. Rossant J. SéGUIN C. A. (2012). Tracing notochord-derived cells using a noto-cre mouse: Implications for intervertebral disc development. Dis. Model Mech. 5, 73–82. 10.1242/dmm.008128 22028328 PMC3255545

[B30] Melrose J. Ghosh P. Taylor T. K. (2001). A comparative analysis of the differential spatial and temporal distributions of the large (aggrecan, versican) and small (decorin, biglycan, fibromodulin) proteoglycans of the intervertebral disc. J. Anat. 198, 3–15. 10.1046/j.1469-7580.2001.19810003.x 11215765 PMC1468186

[B31] Mohanty S. Pinelli R. Pricop P. Albert T. J. Dahia C. L. (2019). Chondrocyte-like nested cells in the aged intervertebral disc are late-stage nucleus pulposus cells. Aging Cell 18, e13006. 10.1111/acel.13006 31290579 PMC6718620

[B32] Newton P. T. Li L. Zhou B. Schweingruber C. Hovorakova M. Xie M. (2019). A radical switch in clonality reveals a stem cell niche in the epiphyseal growth plate. Nature 567, 234–238. 10.1038/s41586-019-0989-6 30814736

[B33] Roberts S. Menage J. Duance V. Wotton S. Ayad S. (1991). 1991 volvo award in basic sciences. Collagen types around the cells of the intervertebral disc and cartilage end plate: An immunolocalization study. Spine (Phila Pa 1976) 16, 1030–1038. 10.1097/00007632-199109000-00003 1948394

[B34] Rutges J. Creemers L. B. Dhert W. Milz S. Sakai D. Mochida J. (2010). Variations in gene and protein expression in human nucleus pulposus in comparison with annulus fibrosus and cartilage cells: Potential associations with aging and degeneration. Osteoarthr. Cartil. 18, 416–423. 10.1016/j.joca.2009.09.009 19833252

[B35] Satija R. Farrell J. A. Gennert D. Schier A. F. Regev A. (2015). Spatial reconstruction of single-cell gene expression data. Nat. Biotechnol. 33, 495–502. 10.1038/nbt.3192 25867923 PMC4430369

[B36] Shichino S. Ueha S. Hashimoto S. Ogawa T. Aoki H. Bin W. 2021. TAS-seq: A robust and sensitive amplification method for beads-based scRNA-seq. bioRxiv. 10.1038/s42003-022-03536-0 PMC9245575 35760847

[B37] Shichino S. Ueha S. Hashimoto S. Ogawa T. Aoki H. Wu B. (2022). TAS-Seq is a robust and sensitive amplification method for bead-based scRNA-seq. Commun. Biol. 5, 602. 10.1038/s42003-022-03536-0 35760847 PMC9245575

[B38] St-Jacques B. Hammerschmidt M. Mcmahon A. P. (1999). Indian hedgehog signaling regulates proliferation and differentiation of chondrocytes and is essential for bone formation. Genes Dev. 13, 2072–2086. 10.1101/gad.13.16.2072 10465785 PMC316949

[B39] Stuart T. Butler A. Hoffman P. Hafemeister C. Papalexi E. Mauck W. M. (2019). Comprehensive integration of single-cell data. Cell 177, 1888–1902. 10.1016/j.cell.2019.05.031 31178118 PMC6687398

[B40] Sugimoto M. Tada Y. Shichino S. Koyamatsu S. Tsumaki N. Abe K. (2022). Universal surface biotinylation: A simple, versatile and cost-effective sample multiplexing method for single-cell RNA-seq analysis. DNA Res. 29, dsac017. 10.1093/dnares/dsac017 35652718 PMC9202638

[B41] Tessier S. Risbud M. V. (2021). Understanding embryonic development for cell-based therapies of intervertebral disc degeneration: Toward an effort to treat disc degeneration subphenotypes. Dev. Dyn. 250, 302–317. 10.1002/dvdy.217 32564440

[B42] Vortkamp A. Lee K. Lanske B. Segre G. V. Kronenberg H. M. Tabin C. J. (1996). Regulation of rate of cartilage differentiation by Indian hedgehog and PTH-related protein. Science 273, 613–622. 10.1126/science.273.5275.613 8662546

[B43] Wen J. Zhu H. Li X. Huang J. Chen Y. Yang Q. (2022). Transforming growth factor-β1 induces transformation of rat meningeal fibroblasts into myofibroblasts by upregulating Shh signaling. Nan Fang. Yi Ke Da Xue Xue Bao 42, 250–255. 10.12122/j.issn.1673-4254.2022.02.12 35365450 PMC8983365

[B44] Zhang F. Hao M. Jin H. Yao Z. Lian N. Wu L. (2017). Canonical hedgehog signalling regulates hepatic stellate cell-mediated angiogenesis in liver fibrosis. Br. J. Pharmacol. 174, 409–423. 10.1111/bph.13701 28052321 PMC5301045

