# Supplementary material for: Chondrocyte-like cells in nucleus pulposus and articular chondrocytes have similar transcriptomic profiles and are paracrine-regulated by hedgehog from notochordal cells and subchondral bone
Source: Front Cell Dev Biol. 2023 May 15;11:1151947. doi: 10.3389/fcell.2023.1151947 (PMC10225674; doi:10.3389/fcell.2023.1151947)
Supplement: Supplementary file 2 [file Image1.PDF]

Supplemental Table 1. Samples used for scRNA-seq analysis

Sample	Description	Monkey	Age (years old)	Sex	GEO deposition
NP1	L4/5	#1	3	male	GSE197380
NP2	L5/6	#1	3	male	GSE197380
NP3	L3/4	#2	3	male	This study
NP4	L3/4	#3	3	male	GSE197380
AC1	femoral condyle	#1	3	male	GSE197380
AC2	femoral condyle	#1	3	male	GSE197380
AC3	femoral condyle	#2	3	male	This study
AC4	femoral condyle	#3	3	male	This study

Supplemental Figure 1

A

total reads	4113413639
reads after quality filtering and adapter trimming	3777011814
mapped reads against Refseq RNA	2300646790
mapping rate against Resfeq RNA (%)	60.91
mapping rate against Resfeq RNA & Cell barcode (%)	78.65
final assigned reads	1809373649
Survived cells	5723
Mean reads per cell	275163
Median symbols od the cell	3738
total genes detected	13756

B

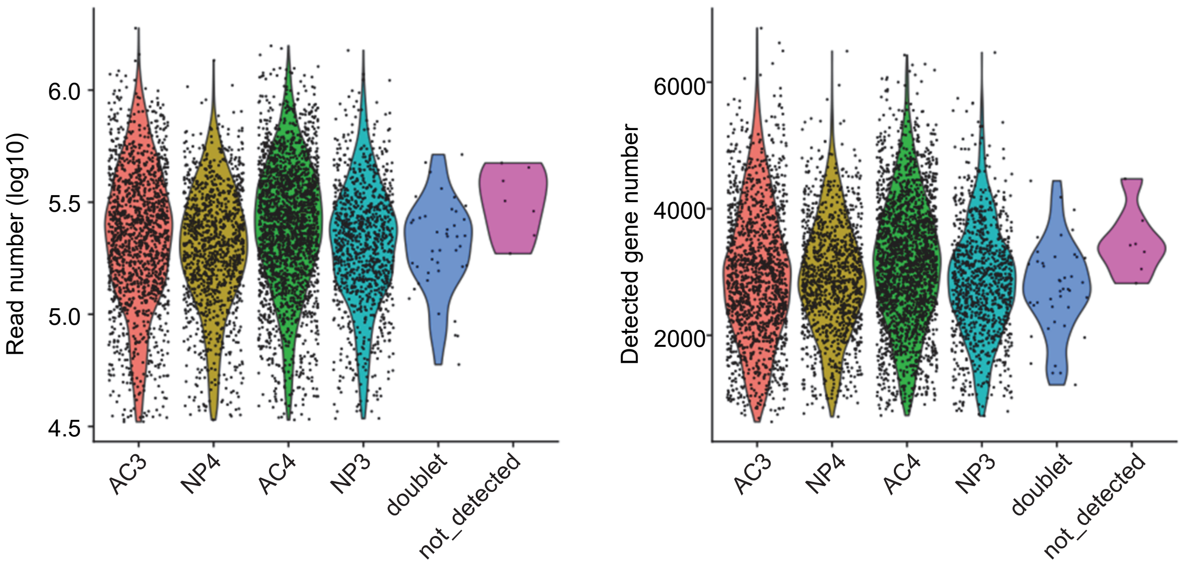

Supplemental Figure 1. Quality control metrics of scRNA-seq data obtained in this study (Monkeys #2 and #3).

(A) Sequencing reads were processed and aligned to the genome, percentage of alignment, number of genes detected per cell and reads per cell. (B) Violin plots showing the numbers of reads and gene numbers detected in cells which were annotated to each sample, “doublet”, and “not detected”.

Supplemental Figure 2

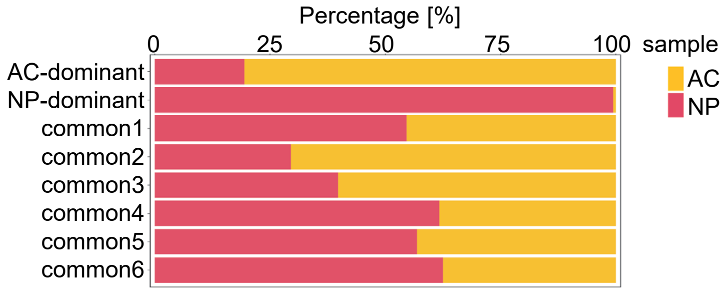

Supplemental Figure 2. The fraction of samples in each cluster adjusted for equal cell number of samples.

Supplemental Figure 3

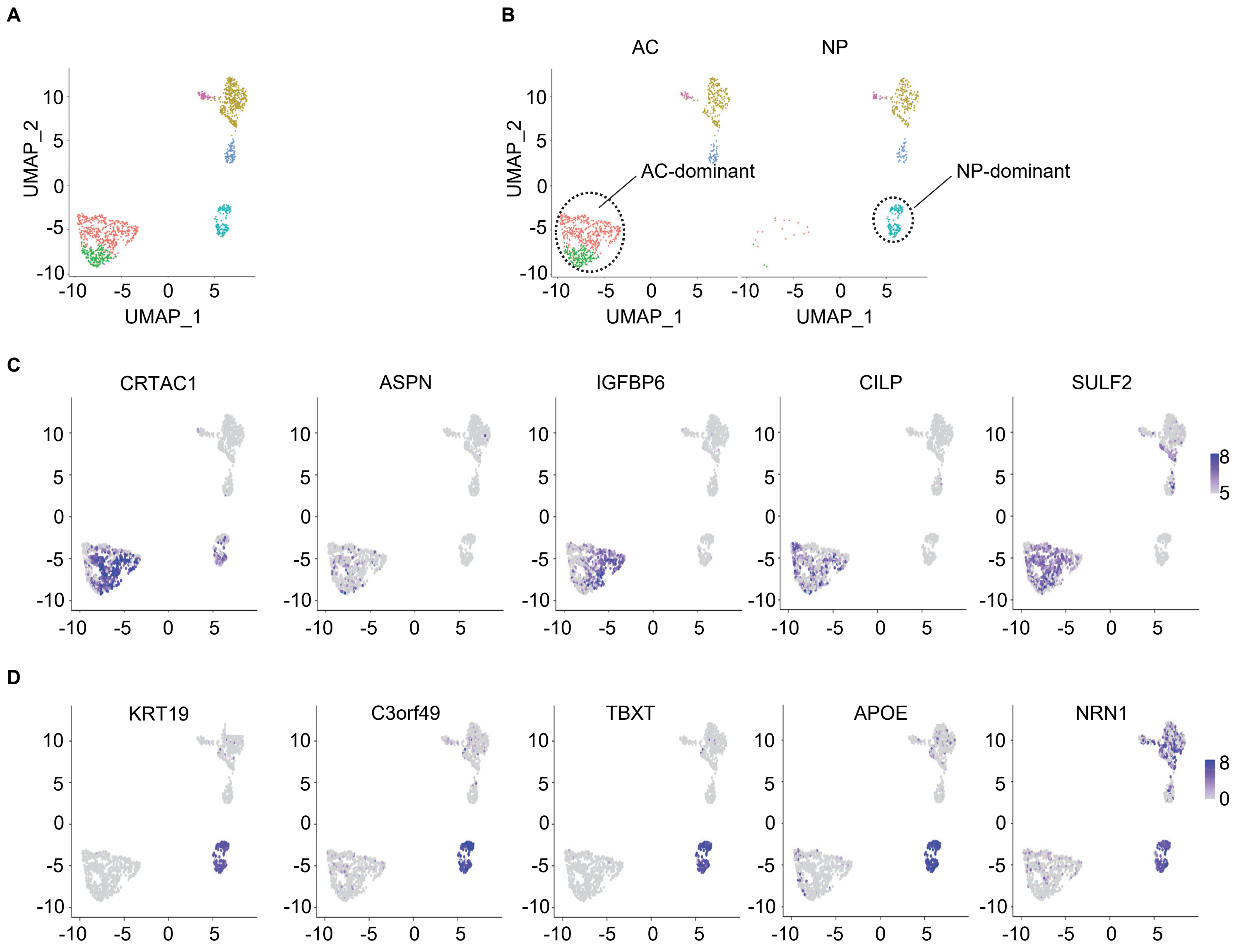

Supplemental Figure 3. scRNA-seq data were analyzed by each monkey. Results from Monkey #1.
(A) UMAP plot for AC and the NP. (B) UMAP plot for AC or NP samples. (C) Feature plots of marker genes for the AC-dominant cluster. (D) Feature plots of marker genes for the NP-dominant cluster.

Supplemental Figure 4

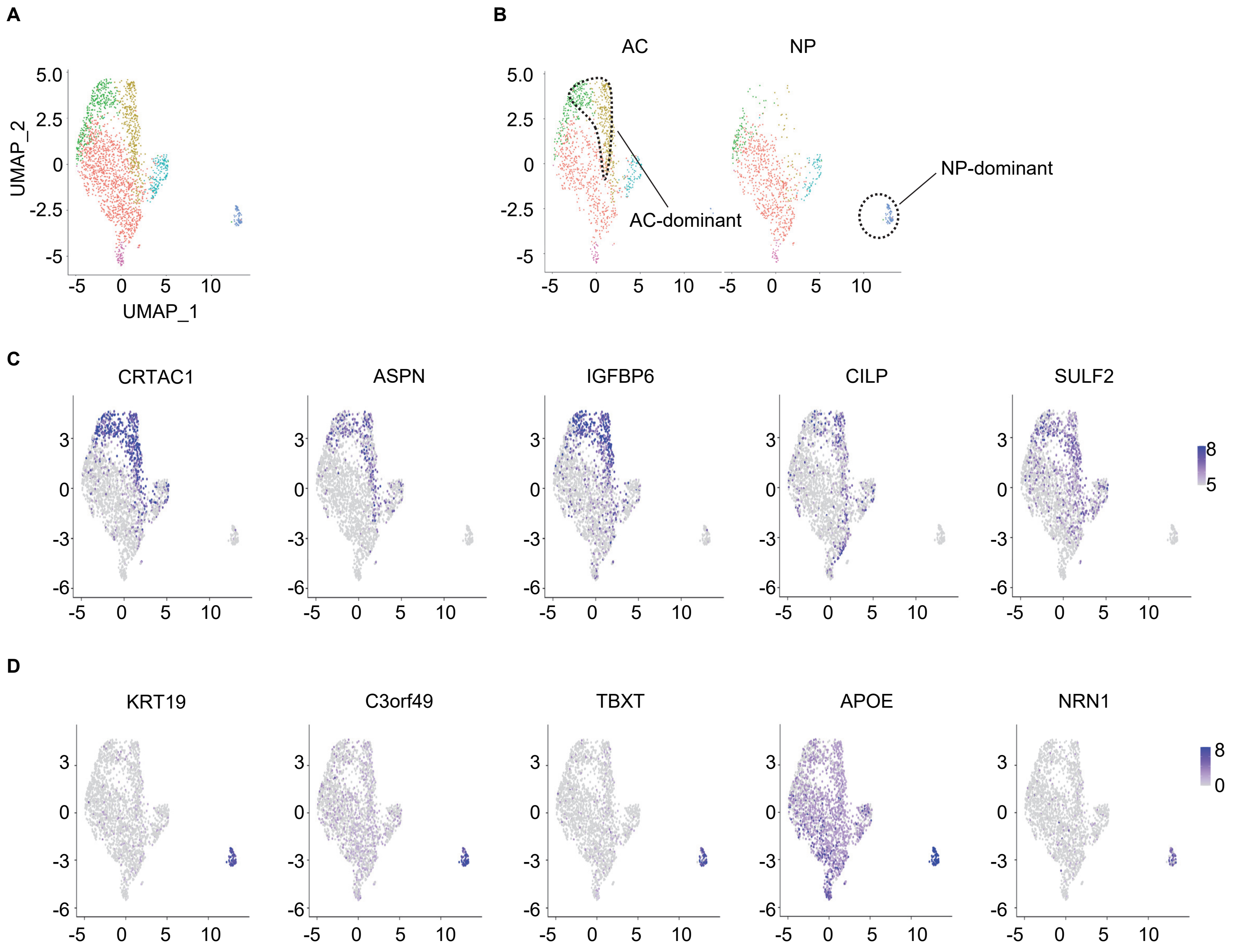

Supplemental Figure 4. scRNA-seq data were analyzed by each monkey. Results from Monkey #2.
(A) UMAP plot for AC and the NP. (B) UMAP plot for AC or NP samples. (C) Feature plots of marker genes for the AC-dominant cluster. (D) Feature plots of marker genes for the NP-dominant cluster.

Supplemental Figure 5

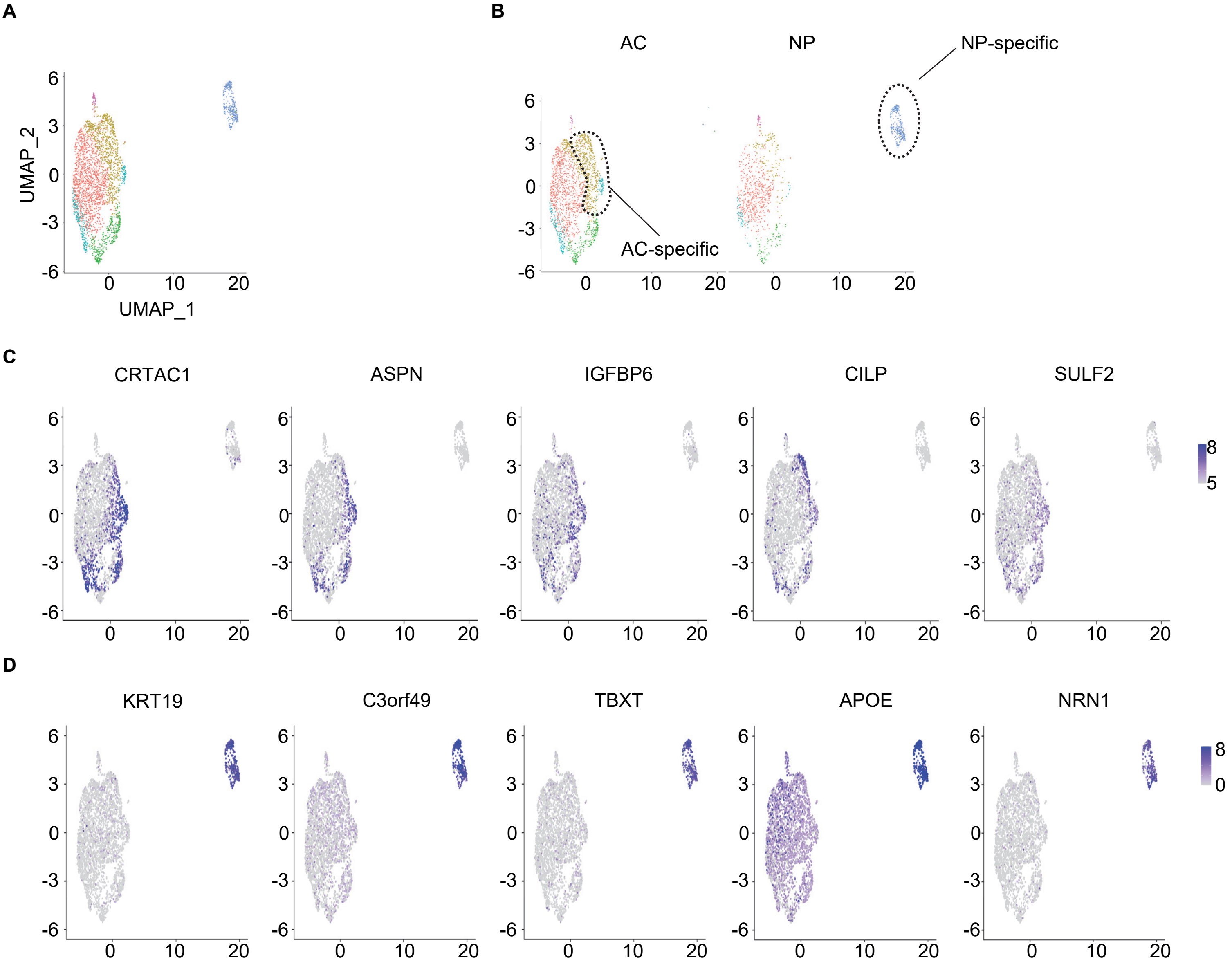

Supplemental Figure 5. scRNA-seq data were analyzed by each monkey. Results from Monkey #3.
(A) UMAP plot for AC and the NP. (B) UMAP plot for AC or NP samples. (C) Feature plots of marker genes for the AC-dominant cluster. (D) Feature plots of marker genes for the NP-dominant cluster.

Supplemental Figure 6

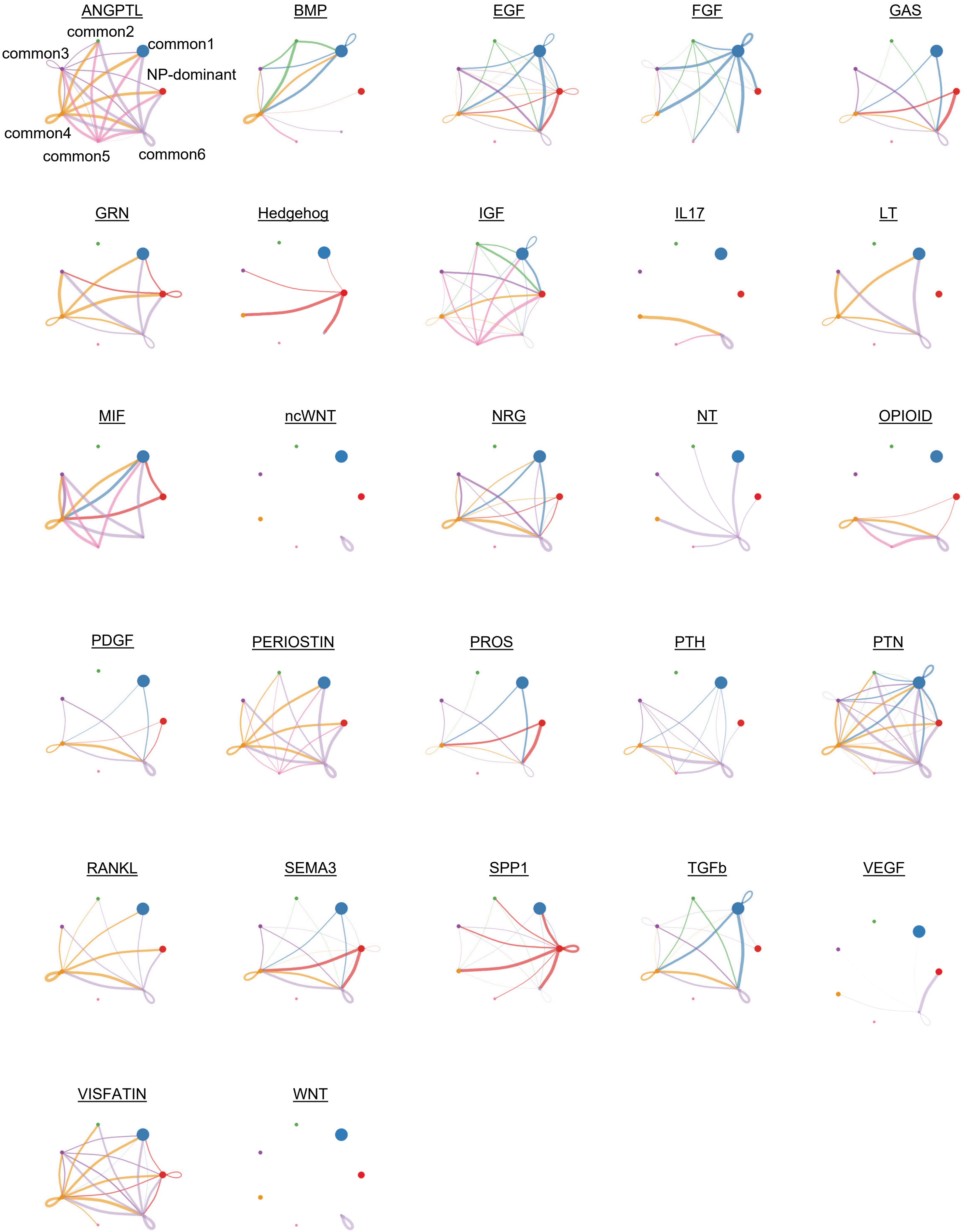

Supplemental Figure 6. Intercellular signaling between clusters identified by CellChat analysis in the NP.

Supplemental Figure 7

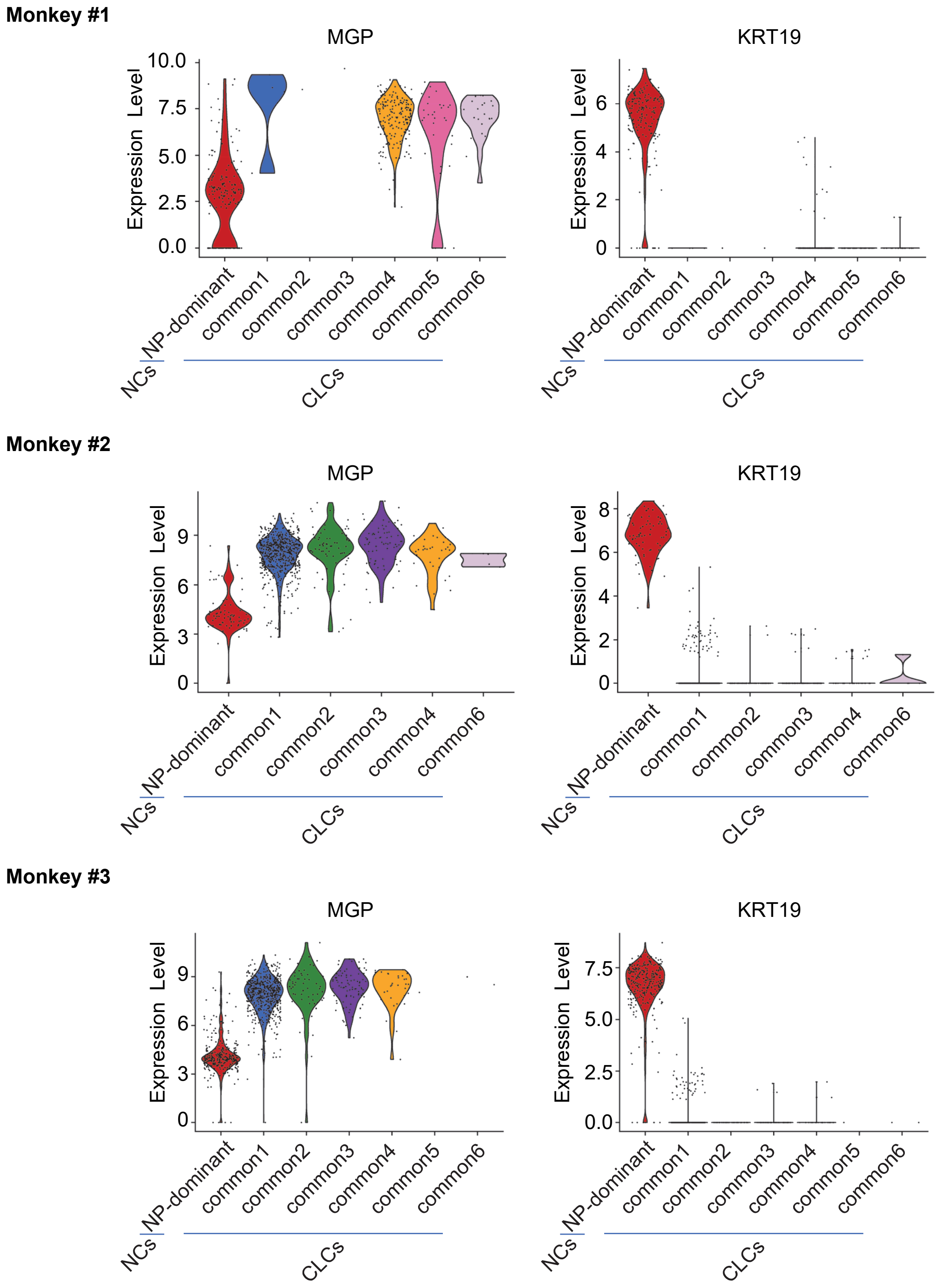

Supplemental Figure 7. Violin plots showing the expression levels of marker genes for CLCs (*MGP*) and NCs (*KRT19*) in the NP sample of each monkey.

Supplemental Figure 8

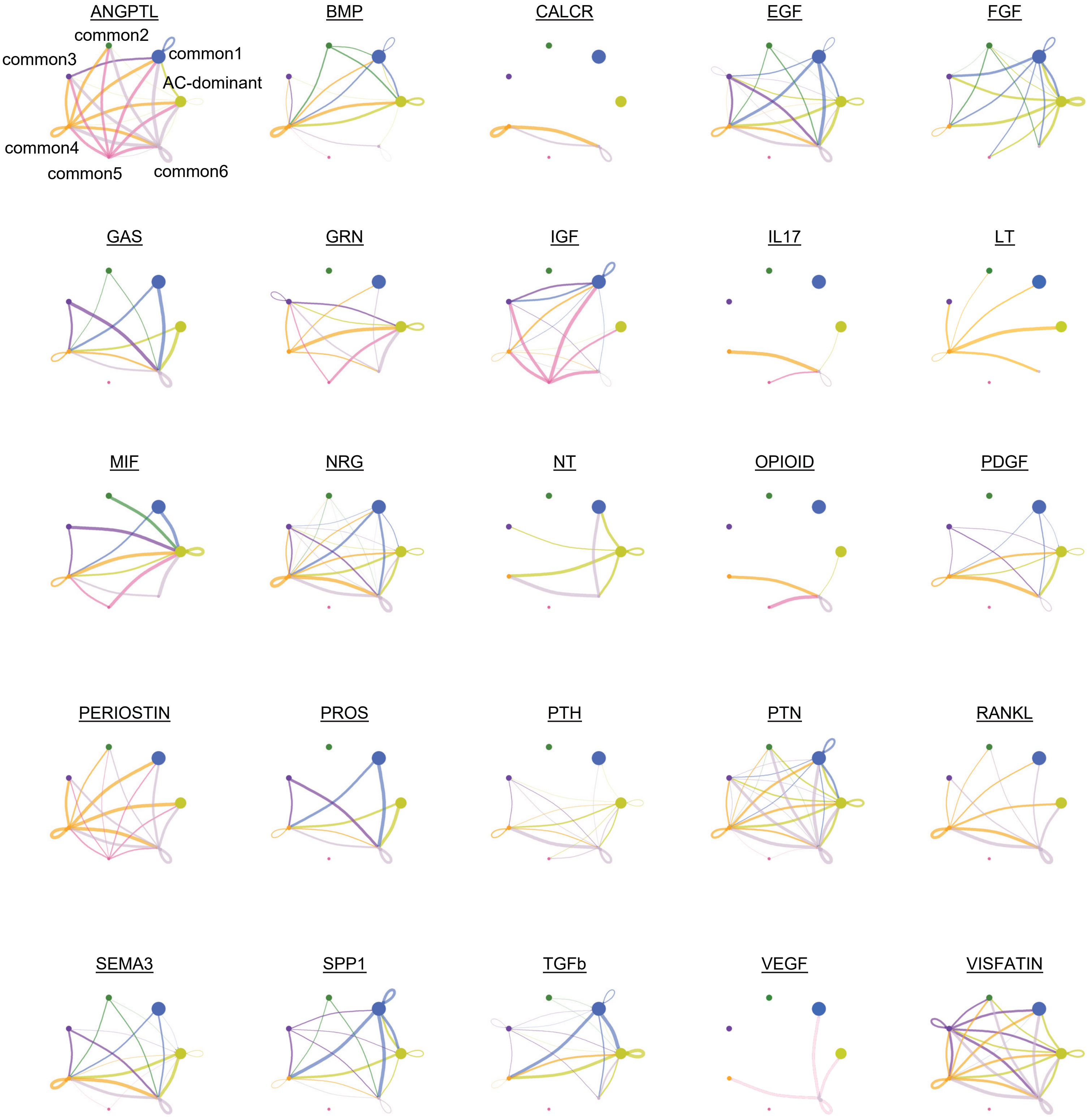

Supplemental Figure 8. Intercellular signaling between clusters in the AC identified by CellChat analysis.

Supplemental Figure 9

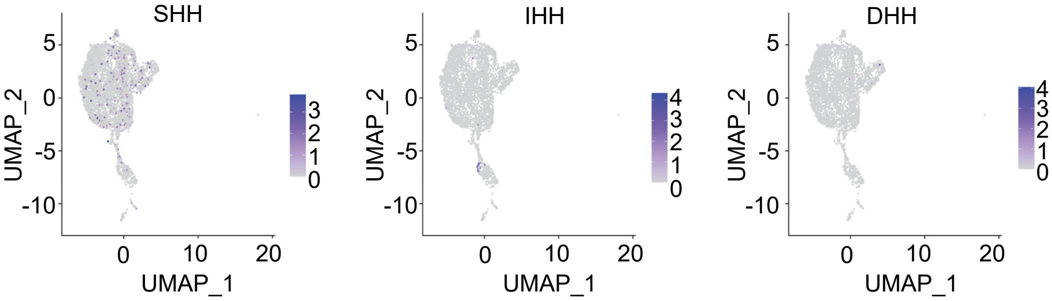

Supplemental Figure 9. Feature plot showing expression of hedgehog family members in AC samples.

Supplemental Figure 10

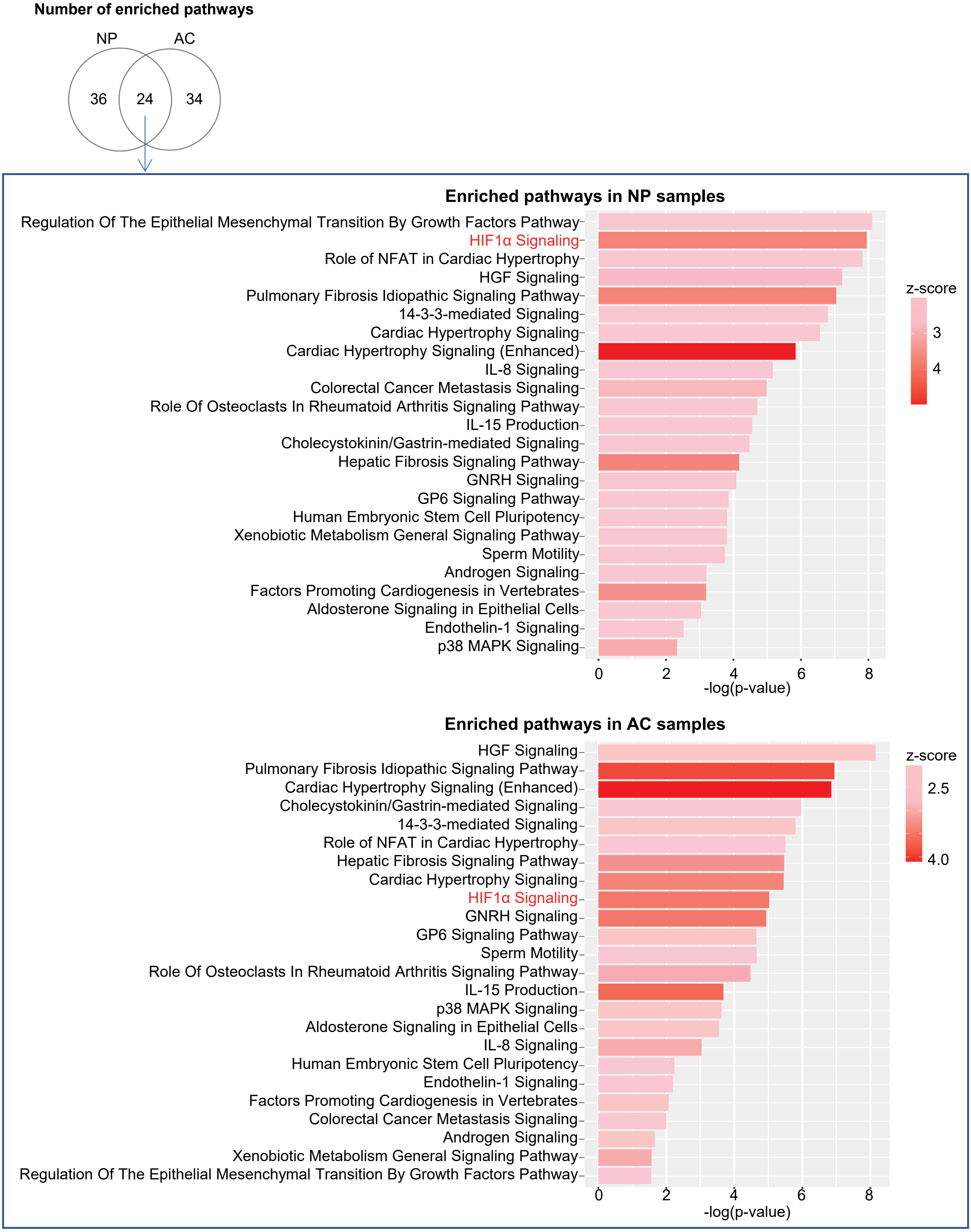

Supplemental Figure 10. Detection of signaling pathways located downstream to hedgehog in the AC and NP.
We identified DEGs between common clusters that appeared to receive hedgehog signaling (common1,3,4, and 6) and those that did not (common2 and 5) and subjected them to IPA in AC and NC samples independently. *Top*, Venn diagram of numbers of pathways enriched in the AC or NP. *Bottom*, 24 pathways that were significantly enriched in both the AC and NP samples are shown.

Supplemental Figure 11

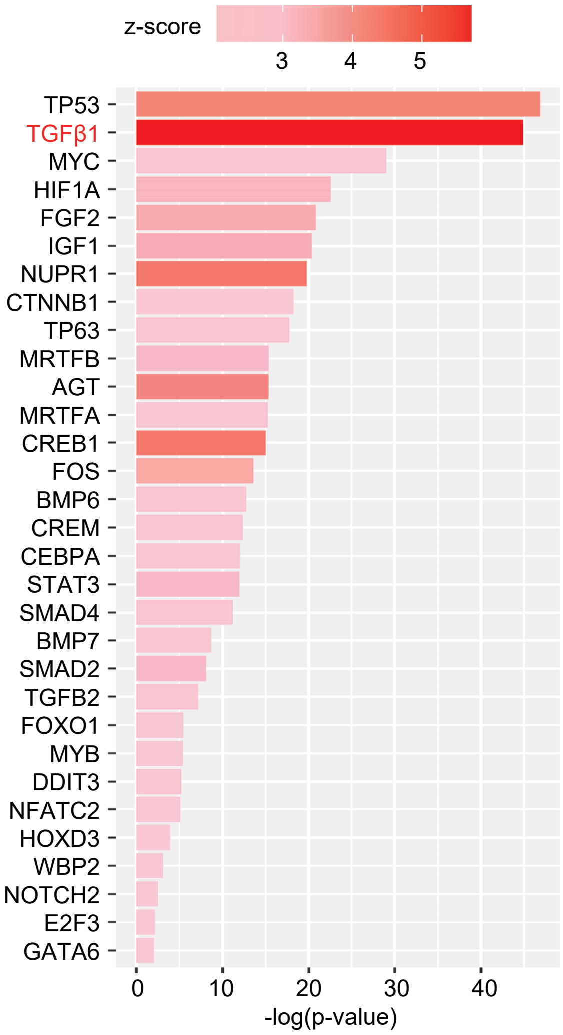

Supplemental Figure 11. Upstream regulator analysis of NCs.
Transcription regulators and growth factors enriched in the NC1 cluster in the analysis of NCs.
